# Beyond main effects of gene‐sets: harsh parenting moderates the association between a dopamine gene‐set and child externalizing behavior

**DOI:** 10.1002/brb3.498

**Published:** 2016-05-31

**Authors:** Dafna A. Windhorst, Viara R. Mileva‐Seitz, Ralph C. A. Rippe, Henning Tiemeier, Vincent W. V. Jaddoe, Frank C. Verhulst, Marinus H. van IJzendoorn, Marian J. Bakermans‐Kranenburg

**Affiliations:** ^1^ Centre for Child and Family StudiesLeiden UniversityLeidenThe Netherlands; ^2^ The Generation R Study GroupErasmus University Medical CenterRotterdamThe Netherlands; ^3^ Department of Child and Adolescent PsychiatryErasmus University Medical Center‐Sophia Children′s HospitalRotterdamThe Netherlands; ^4^ Department of EpidemiologyErasmus University Medical CenterRotterdamThe Netherlands; ^5^ Department of PsychiatryErasmus University Medical CenterRotterdamThe Netherlands; ^6^ Department of PediatricsErasmus University Medical CenterRotterdamThe Netherlands; ^7^ School of Pedagogical and Educational SciencesErasmus UniversityRotterdamThe Netherlands

**Keywords:** Behavior problems dopaminergic system, child development, dopamine, externalizing behavior, gene‐environment interaction, gene‐set, harsh parenting, parenting, preschoolers

## Abstract

**Background:**

In a longitudinal cohort study, we investigated the interplay of harsh parenting and genetic variation across a set of functionally related dopamine genes, in association with children's externalizing behavior. This is one of the first studies to employ gene‐based and gene‐set approaches in tests of Gene by Environment (G × E) effects on complex behavior. This approach can offer an important alternative or complement to candidate gene and genome‐wide environmental interaction (GWEI) studies in the search for genetic variation underlying individual differences in behavior.

**Methods:**

Genetic variants in 12 autosomal dopaminergic genes were available in an ethnically homogenous part of a population‐based cohort. Harsh parenting was assessed with maternal (*n* = 1881) and paternal (*n* = 1710) reports at age 3. Externalizing behavior was assessed with the Child Behavior Checklist (CBCL) at age 5 (71 ± 3.7 months). We conducted gene‐set analyses of the association between variation in dopaminergic genes and externalizing behavior, stratified for harsh parenting.

**Results:**

The association was statistically significant or approached significance for children without harsh parenting experiences, but was absent in the group with harsh parenting. Similarly, significant associations between single genes and externalizing behavior were only found in the group without harsh parenting. Effect sizes in the groups with and without harsh parenting did not differ significantly. Gene‐environment interaction tests were conducted for individual genetic variants, resulting in two significant interaction effects (rs1497023 and rs4922132) after correction for multiple testing.

**Conclusion:**

Our findings are suggestive of G × E interplay, with associations between dopamine genes and externalizing behavior present in children without harsh parenting, but not in children with harsh parenting experiences. Harsh parenting may overrule the role of genetic factors in externalizing behavior. Gene‐based and gene‐set analyses offer promising new alternatives to analyses focusing on single candidate polymorphisms when examining the interplay between genetic and environmental factors.

## Introduction

Young children with externalizing problem behaviors, including oppositional behavior, aggression, and attention problems, are at increased risk for problems later in life. Such problems include poor school performance, peer‐relationship issues, aggression, violence, and crime (Campbell et al. 2000, 2006; Reef et al. 2011). Child externalizing behavior is predicted both by child factors, including genetics, and by environmental factors, including parenting behavior (e.g., Haberstick et al. 2005; Miner and Clarke‐Stewart 2008; Saudino et al. 2008). The effects of these factors are not independent of one another. Significant progress has been made in understanding how environmental factors interact with genetic factors to affect child development (Moffitt et al. 2005; Rutter 2006; Ellis et al. 2011), but the genetic factors are usually considered independently in analyses. In this study we examine how harsh parenting interacts with genetic variation across multiple child dopamine genes in shaping child externalizing behavior. This is a novel approach to the study of gene‐environment interactions (G × E), as it takes into account genetic variation across whole genes and gene‐sets (a combination of related genes). This approach complements the more conventional analyses at the level of single‐nucleotide polymorphisms (SNPs).

The pursuit of genetic underpinnings of complex child problem behaviors, including externalizing behavior and aggression, has yielded limited success. Longitudinal twin studies of childhood aggression – an overlapping phenotype with externalizing behavior – show moderate to high estimates of heritability (51–72%) (Hudziak et al. 2003). These findings are supported by SNP heritability estimates from genome‐wide complex trait analysis (GCTA) for externalizing and other behavior problems in children, conducted in two population‐based Dutch cohorts (Pappa et al. 2015a). Common polymorphisms in several candidate genes have been reported in association with childhood aggression and externalizing (Volavka et al. 2004; Oades et al. 2008; Takahashi et al. 2012), but these effects have not held up after meta‐analysis (Vassos et al. 2014). The candidate gene approach has been generally criticized for nonreplication and potential false discovery, related to publication bias for positive findings, inadequate power to detect true effects, and improper correction for multiple testing (Tabor et al. 2002; Sullivan 2007). Furthermore, it has been argued that variation in complex traits such as externalizing behavior is not readily attributable to variation at single genetic loci or on single genes, but rather to variation at multiple genetic loci across the genome (Reif and Lesch 2003). Thus, the lack of robust effects in past candidate gene studies might be partially owing to the fact that externalizing behavior is a complex phenotype, and likely has a polygenic nature in humans, as has been demonstrated previously in mice for aggressive behavior (Brodkin et al. 2002). Expanding the search for genetic correlates of aggression‐related phenotypes, including externalizing behavior, in children and adults via genome‐wide association studies (GWAS) has allowed testing for associations between millions of SNPs across the genome and the outcome without an a priori hypothesis. These types of studies treat all SNPs independently, and have provided only suggestive evidence for a genetic main effect on externalizing phenotypes (e.g., Dick et al. 2004; Tielbeek et al. 2012; Viding et al. 2013; Pappa et al. 2015b).

One of the factors that can bridge the gap between the substantial heritability estimates and the lack of individual genetic associations is the environment. Genetic effects are now widely accepted as being open to moderation by environmental influences. Until recently, most studies investigating G × E have focused on specific polymorphisms within candidate genes with functions implicated in the outcome of interest (Manuck and McCaffery 2014), although candidate G × E studies received similar critiques as studies searching for main effects (Duncan and Keller 2011; Dick et al. 2015). Given the polygenic nature of complex phenotypes, consideration of the moderating effects of environment at a multigene level becomes important. Candidate genes studied in the context of parenting and externalizing behavior are often related to the dopamine system, a neurotransmitter system involved in motivation and reward processing, and in the regulation of emotion, action, and attention (Robbins and Everitt 1999; Keltikangas‐Järvinen and Salo 2009). For instance, polymorphisms on the dopamine receptor D2 (DRD2), D4 (DRD4), and dopamine transporter gene (DAT) genes reportedly moderate the association between parenting and child problem behavior (e.g., Keltikangas‐Järvinen and Salo 2009; Bakermans‐Kranenburg and Van IJzendoorn 2011; Windhorst et al. 2015). Such candidate genes can be considered indicators of underlying genetic pathways.

In contrast to hypothesis‐based candidate gene studies, genome‐wide environmental interaction (GWEI) studies investigate interaction effects between individual SNPs located across the genome and environmental factors on an outcome, in a hypothesis‐free way similar to GWAS studies (Aschard et al. 2012). Genome‐wide analyses require extremely large samples because the high numbers of SNPs tested require conservative corrections for multiple testing. As a consequence, it is difficult to detect small effects of individual SNPs (Hirschhorn and Daly 2005), particularly on complex traits. Moreover, in very large samples the careful assessment of environmental data is complicated due to practical issues (Khoury and Wacholder 2009).

These challenges impede G × E research and highlight the need for new methods. One approach used in the search for genetic main effects on behavioral outcomes aggregates genetic variance of *many* SNPs within a single gene or within a predefined set of genes that includes multiple functionally or biologically related genes (e.g., Fridley and Biernacka 2011; Lips et al. 2012; Winham and Biernacka 2013). Testing joint effects of multiple SNPs greatly reduces multiple testing, and consequently improves power. Thus, SNPs with individually marginal effect sizes could have a significant *combined* effect (e.g., Fridley and Biernacka 2011; Lips et al. 2012; Winham and Biernacka 2013). Another advantage is that the results are biologically interpretable. That is more difficult for individual SNPs, as the functional role of many individual genetic variants remains unknown. Although the approach of testing the joint effect of multiple SNPs within a single gene (gene‐based analysis) or within a set of genes (gene‐set analysis) has so far mostly been used to examine genetic main effects, they also provide new opportunities for studying G × E. Unfortunately, most statistical programs do not yet provide an option to include estimations for gene‐environment interaction effects (Winham and Biernacka 2013). Several other approaches have arisen over the years to go beyond single candidate genes in G × E research, for example, by assessing interaction effects of the environment and polymorphisms of multiple (unrelated) candidate genes, investigating cumulative‐genetic plasticity to environmental influences (e.g., Belsky and Beaver 2011). The approach used in this study is different, as we tested the joint effect of multiple polymorphisms on genes that are biologically related, instead of assessing a cumulative effect of multiple risk‐ or susceptibility alleles. As another alternative, polygenic risk scores can be computed based on SNP weights derived from GWAS studies investigating single SNP associations with the outcome of interest. This approach has also been used in search for gene‐environment interactions (e.g., Salvatore et al. 2015) Although this approach is also promising in unraveling the interplay between genetic and environmental factors underlying complex traits, gene‐set analyses may be of additional use to provide more insight in the biological relevant pathways and mechanisms involved.

In this exploratory study we extend the gene‐based and gene‐set approaches to include G × E, using a tool for gene‐based and gene‐set analyses: Joint Association of Genetic Variants (JAG) (http://ctglab.nl/software/jag, Lips et al. (2015). We examine the interplay between harsh parenting and variation across children's dopamine genes in association with externalizing behavior, in a large ethnically homogenous subsample of a population‐based cohort study. We assess aggregated genetic variation across a “set” of multiple dopamine genes. Because the inclusion of an environmental moderator (presence or absence of harsh parenting) in this gene‐set analysis is novel and exploratory, we can only speculate about a directional hypothesis. On the one hand, aggregated genetic variation might have strongest effects in high‐risk environments (harsh parenting), which is consistent with the hypothesis that negative environments “trigger” risk alleles (Shanahan and Hofer 2005). On the other hand, aggregated genetic variance might have strongest effects in low‐risk environments (no harsh parenting) in which environmental homogeneity allows for more genetically driven phenotypic differentiation. This is one of the first studies to employ a gene‐set analysis in tests of G × E effects on complex behavior. This approach can offer an important alternative or complement to candidate gene and GWEI studies in the search for genetic variation underlying individual differences in behavior.

## Method

### Setting

The current investigation is embedded in the Generation R Study, a prospective cohort study investigating development from fetal life into young adulthood in Rotterdam, the Netherlands (Jaddoe et al. 2012; Tiemeier et al. 2012). Briefly, all pregnant women living in Rotterdam with an expected delivery date between April 2002 and January 2006 were invited to participate. The study has been approved by the Medical Ethical Committee of the Erasmus Medical Center, Rotterdam. Written informed consent was obtained from all adult participants.

### Study population

Genetic information and questionnaire data at child age 3 (harsh parenting) and 5 (CBCL) were included in the analyses. We report separate analyses for harsh parenting by the father and by the mother. Genetic data were available for 2830 Caucasian children, for whom paternal and maternal harsh parenting data were available for 1817 and 2005 children, respectively. If data were available for multiple siblings, one child per sibling pair was randomly selected and included in the analyses. The final numbers for paternal and maternal harsh parenting consisted of 1710 and 1881 children, respectively. Sample characteristics are presented in Table 1. The groups with and without harsh parenting differed on a number of characteristics, including gender, parity, birth weight, age of the mother, maternal and paternal educational level, depressive and anxiety symptoms of mother and father, and child externalizing behavior.

**Table 1:** Sample characteristics

Group	Paternal harsh parenting				Maternal harsh parenting			
	Total	Without harsh parenting	With harsh parenting	Significance	Total	Without harsh parenting	With harsh parenting	Significance
*n*	1710	1113	597		1881	1208	673	
Child characteristics								
Gender (% boys)	50.40	46.00	58.60	**	51.10	49.50	54.10	
Parity (% primiparous)	62.20	64.00	58.80	*	61.60	63.40	58.30	*
Birth weight	3563.11 ± 520.89	3542.45 ± 507.73	3601.63 ± 542.91	*	3553.58 ± 525.39	3539.25 ± 519.93	3579.30 ± 534.48	
Child age at CBCL completion	71.21 ± 3.65	71.16 ± 3.75	71.29 ± 3.47		71.21 ± 3.55	71.13 ± 3.43	71.35 ± 3.74	
CBCL sum score externalizing behavior	6.70 ± 5.98	5.79 ± 5.31	8.40 ± 6.75	**	6.76 ± 6.00	5.90 ± 5.45	8.31 ± 6.61	**
Parent characteristics								
Age mother at intake	32.13 ± 3.77	32.04 ± 3.84	32.30 ± 3.63		32.14 ± 3.84	32.28 ± 3.80	31.89 ± 3.91	*
Age father at intake	34.15 ± 4.72	34.18 ± 4.82	34.08 ± 4.55		34.14 ± 4.71	34.15 ± 4.55	34.10 ± 5.00	
Educational level mother (% high)	77.80	78.00	77.20		77.00	78.20	74.00	*
Educational level father (% high)	75.30	75.90	74.30		73.70	76.40	68.90	**
Marital status mother/father (% married/living together)	95.10	94.70	95.80		93.70	93.50	93.90	
Maternal smoking during pregnancy (% never smoked during pregnancy)	80.90	82.30	78.40		80.30	80.50	80.00	
Maternal drinking during pregnancy (% never drunk during pregnancy)	24.80	24.70	24.90		25.40	25.90	24.50	
Depressive symptoms mothers BSI	0.09 ± 0.21	0.08 ± 0.20	0.11 ± 0.24	*	0.09 ± 0.22	0.08 ± 0.21	0.11 ± 0.23	**
Depressive symptoms fathers BSI	0.08 ± 0.20	0.05 ± 0.17	0.10 ± 0.24	**	0.07 ± 0.20	0.07 ± 0.18	0.09 ± 0.23	
Anxiety symptoms mothers BSI	0.15 ± 0.25	0.14 ± 0.24	0.18 ± 0.27	**	0.16 ± 0.26	0.14 ± 0.23	0.19 ± 0.30	**
Anxiety symptoms fathers BSI	0.15 ± 0.24	0.13 ± 0.22	0.18 ± 0.26	**	0.15 ± 0.24	0.14 ± 0.23	0.16 ± 0.26	
Harsh parenting mother (sum score 5 items)					0.73 ± 1.28	0.00 ± 0.00	2.03 ± 1.40	**
Harsh parenting father (sum score 5 items)	0.70 ± 1.22	0.00 ± 0.00	2.00 ± 1.28	**				

Sample characteristics are presented for paternal harsh parenting and maternal harsh parenting (total group, group without harsh parenting, and group with harsh parenting). The number of missing values varies across variables. Unless otherwise specified, numbers in the table represent mean ± SD. All percentages are valid percentages. Using t‐tests and chi‐square tests, all variables were compared between the groups with and without harsh parenting.

*P < 0.05, **P < 0.01.

Nonresponse analyses were performed to compare the children with parenting data to the children with missing parenting data. Compared to excluded children, included children had on average a higher birth weight, were more often primiparous, and were younger at CBCL assessment. The mothers of these children and their partners were older at intake, educational level of the mother and partner was higher, parents were more often married or living together, fewer mothers smoked but more mothers drank alcohol during pregnancy. Mothers of included children had higher depressive and anxiety symptom scores compared to mothers of excluded children.

### Measures

#### Genetic data

##### Genotyping of GWAS data

DNA was collected from cord blood samples at birth. All samples were genotyped using Illumina Infinium II HumanHap610 Quad Arrays (Illumina, San Diego, CA, USA) following standard manufacturer's protocols. Some children (320), for whom DNA was missing, provided blood samples at the age of 6, and these samples were genotyped using the Human 660 Quad Arrays of Illumina. Intensity files were analyzed using the Beadstudio Genotyping Module software v.3.2.32 and genotype calling based on default cluster files. Samples displaying call rates below 95% and mismatch between called and phenotypic gender were excluded. In addition, individuals identified as genetic outliers by the identity‐by‐state (IBS) clustering analysis (>4 standard deviations away from the HapMap CEU population mean) were considered of other ancestry and were excluded from the analyses. Finally, genotyped SNPs with minor allele frequency (MAF) < 0.01, SNP Call Rate <0.98 and Hardy–Weinberg Equilibrium (HWE) *P*‐value <1 × 10^−6^ were filtered. SNPs that showed differential missingness between arrays were excluded (for additional information on GWAS data in the Generation R study, see Medina‐Gomez et al. 2015). GWAS data were available for 2830 Caucasian children. The number of genotyped SNPs (build 37) was 489,878.

##### Gene‐set selection and SNP assignment

On the basis of a wide literature exploring associations between dopamine genes and behavior (e.g., Talkowski et al. 2007; Gardner et al. 2008; Nemoda et al. 2011; Gorwood et al. 2012), we selected a gene‐set of autosomal dopaminergic genes: DRD1, DRD2, DRD3, DRD4, DRD5, COMT, DBH, DDC, DAT, TH, DARPP‐32, VMAT1, VMAT2, ANKK1 (Table 2). All genotyped SNPs that passed quality control were mapped to genes on the basis of National Center for Biotechnology Information (NCBI) human assembly build 37 (Genome Reference Consortium GRCh37, dbSNP release 131, UCSC assembly hg 19). The SNP‐to‐gene annotation was performed using the software package JAG (Lips et al. 2015). No flanker regions were specified, that is, the transcription start site and transcription end site were used as boundaries of the genes. The Illumina Array did not yield genotyped SNPs on the DRD4, DRD5, and DARPP‐32 genes. The DRD4 48 bp variable number of tandem repeats polymorphism (VNTR), however, was further individually genotyped (see Appendix S1), and this polymorphism was (manually) added to the GWAS data. The final gene‐set consisted of 12 genes including 151 genetic variants. Minor allele frequencies (MAFs) of these genetic variants are presented in Appendix S2.

**Table 2:** Dopamine genes in the gene‐set

Gene	Alias	Entrez GeneID	Gene name	Chromosome number	Transcription start site (bp)	Transcription end site (bp)	Strand	Number of SNPs available in GWAS data
DRD1		1812	Dopamine D1 receptor	5	174,867,675	174,871,163	−	2
DRD2		1813	Dopamine D2 receptor	11	113,280,317	113,346,001	−	15
DRD3		1814	Dopamine D3 receptor	3	113,847,557	113,897,899	−	12
DRD4		1815	Dopamine D4 receptor	11	637,305	640,706	+	0 (but 1 VNTR manually added)
DRD5		1816	Dopamine D5 receptor	4	9,783,258	9,785,633	+	0
DAT	SLC6A3, DAT1	6531	Dopamine Transporter	5	1,392,905	1,445,543	−	13
VMAT1	SLC18A1	6570	Vesicular monoamine transporter 1 (VMAT1)	8	20,002,366	20,040,717	−	20
VMAT2	SLC18A2	6571	Vesicular monoamine transporter 2 (VMAT2)	10	119,000,584	119,038,941	+	18
TH		7054	Tyrosine hydroxylase	11	2,185,159	2,193,035	−	2
DDC		1644	Dopa decarboxylase	7	50,526,134	50,633,154	−	32
COMT		1312	Catechol‐*O*‐methyltransferase	22	19,929,263	19,957,498	+	14
DARPP‐32	PP1R1B	84152	Dopamine‐ and cAMP‐regulated phosphoprotein, 32 kDa	17	37,783,177	37,792,878	+	0
DBH		1621	Dopamine beta‐hydroxylase	9	136,501,485	136,524,466	+	17
ANKK1		255239	Ankyrin repeat and kinase domain containing 1	11	113,258,513	113,271,140	+	5

Gene locations are based on NCBI (National Center for Biotechnology Information) human assembly build 37 (Genome Reference Consortium GRCh37, dbSNP release 131, UCSC assembly hg 19). For DRD5 and DARPP‐32 (marked in gray) no genotyped SNPs were available from Illumina.

#### Harsh parenting

Harsh parenting was assessed with a parental self‐report questionnaire when the child was 3 years old (36 ± 1.2 months). In a previous study of the same cohort (Jansen et al. 2012), six items of the Parent‐Child Conflict Tactics scale (Straus et al. 1998) were selected based on factor analysis to constitute a harsh discipline scale. The scale consisted of the following items: “shook my child”, “shouted or screamed angrily at my child”, “called my child names”, “threatened to give a slap, but I didn't do it”, “angrily pinched my child's arm”, “called my child stupid, lazy, or something like that”. Confirmatory factor analyses indicated good fit for the harsh parenting factor in both mothers and fathers (for additional details, see Jansen et al. 2012). Parents rated their use of discipline behaviors during the past 2 weeks on a six‐point scale ranging from “never” to “five times or more”. The categories twice, three times, four times, and five times were combined because of very low prevalence rates. This resulted in three response categories per item: never (0), once (1), and twice or more (2). A sum score was computed based on the item scores. The prevalence of the item “shouted or screamed angrily at my child” was, however, very high; therefore, this item was excluded from the sum score. Children were assigned to the group with harsh parenting experiences if the mother or father had reported that at least one of the remaining five harsh parenting items had occurred within the last 2 weeks (i.e., sum score ≥1). In the group without harsh parenting experiences, parents had reported that none of the items had occurred within the last 2 weeks (i.e., sum score 0). This was done separately for maternal and paternal harsh parenting. The group sizes of the group without versus with harsh parenting were 1113 versus 597 for paternal harsh parenting, and 1208 versus 673 for maternal harsh parenting. The association between paternal and maternal harsh parenting dichotomies was significant, *Χ* ^*2*^(1) = 123.62 *P *<* *0.001, *φ *= 0.27, *P *<* *0.001. For more information on harsh parenting sum scores and dichotomization, see Appendix S3. Since JAG does not provide an option to include gene‐environment interaction effects, we ran the JAG tool for gene‐based and gene‐set analyses separately in the groups with and without harsh parenting experiences.

#### Externalizing behavior

Externalizing behavior of the child was assessed by parental report when the child was 5 years of age (71 ± 3.7 months). The primary caregiver filled out a Dutch version of the Child Behavior Checklist (CBCL/1.5–5), a widely used questionnaire with 99 items concerning the child's behavior in the previous 2 months. The CBCL for ages 1.5–5 was used for all children, because the majority (66%) of the children were younger than 6 years old at the time of assessment. We considered it important to use only one version of the CBCL to enhance comparability across all children. The 1.5–5 version was chosen because the version for 6‐ to 18‐year‐olds contains questions that are less applicable to 5‐year‐olds, for example, questions concerning smoking tobacco. Each item is scored as 0 = *not* true, 1 = *somewhat or sometimes* true, and 2 = *very true or often* true. Seven empirically based syndrome scales were identified: emotionally reactive, anxious/depressed, somatic complaints, withdrawn, sleep problems, attention problems, and aggressive behavior (Achenbach and Rescorla 2000). The broadband scale “Externalizing” comprises item scores on the subscales aggressive behavior and attention problems scales. Higher scores indicated more problems. Internal consistency of the CBCL was *α *= 0.90. CBCL scores on externalizing behavior were missing for 6.6% of the children for paternal harsh parenting analyses and for 7.3% of the children for maternal harsh parenting analyses. Missing values on externalizing behavior were imputed by regression imputation using as predictors CBCL scores on externalizing behavior collected at 18 and 36 months.

#### Covariates

The following covariates were included in all analyses: age of the child when the CBCL was completed, gender of the child and four ancestry‐informative principal components. These principal components were obtained by principal component analyses of the GWAS data of the Caucasian children (*n* = 2830) and were included to account for population‐specific variations in alleles distribution of the SNPs to control for effects due to population stratification (e.g., Price et al. 2006).

### Analyses

Prior to the main analyses, we ran association tests for all individual SNPs and harsh parenting using PLINK version v1.07 (http://pngu.mgh.harvard.edu/purcell/plink/, Purcell et al. 2007) to check whether allele frequencies of the SNPs differed between the groups with and without harsh parenting experiences, in order to rule out gene‐environment correlations.

#### Gene‐set analyses

Gene‐set analyses were conducted using JAG (Lips et al. 2015). We ran separate analyses for the total group and for the two groups with and without harsh parenting, for paternal and maternal. The first step in gene‐set analysis is to conduct self‐contained tests, which test the null hypothesis that the dopamine gene‐set was not associated with externalizing behavior, by resampling in the current gene‐set only and not by comparing other (unrelated) sets of genes (competitive test; see below). For every self‐contained test, the test statistic was computed using the sum of the −log_10_ of the *P‐value*s for the single SNP associations for all the SNPs in the gene‐set. Empirical *P*‐values were obtained with at least 9000 permutations of the phenotype, keeping linkage disequilibrium and number of SNPs and genes constant. The empirical *P*‐value was calculated as the proportion of test statistics that were equal to or higher than the test statistic of the original data. For the gene‐set analyses, an empirical *P*‐value <0.05 was considered significant. For additional details, see Lips et al. (2015).

In case of a significant empirical *P‐*value in the self‐contained test for the dopamine gene‐set, a competitive test was conducted. Competitive tests show whether the gene‐set of interest is more strongly associated with externalizing behavior than randomly generated gene‐sets matched for the same number of genes. Competitive tests for polygenic traits like externalizing behavior are essential, because associations with a gene‐set that emerge in the self‐contained test can be due to the polygenic background. 150 random control gene‐sets matching the original dopamine gene‐set on number of genes were generated. Self‐contained tests similar to those described above were conducted for each of the control gene‐sets. Finally, a competitive *P‐*value was calculated as the proportion of self‐contained *P‐*values for the random gene‐sets that were lower than the empirical self‐contained *P‐*value for the dopamine‐set. Competitive *P‐*values <0.05 were considered to be significant.

##### Testing the interaction of harsh parenting and the dopamine gene‐set

Joint Association of Genetic Variants currently does not allow for a direct test of the interaction between harsh parenting and genetic variance in the dopamine gene‐set. To test this interaction indirectly, analyses were run on the stratified samples (with and without harsh parenting). Subsequently, the *P*‐values for the groups were statistically compared taking sample sizes into account, using a meta‐analysis tool in R (R version 3.1.2, package “metafor”, R Core Team (2015), http://www.R-project.org/).

##### Computing effect estimates

As JAG does not provide effect estimates, we computed Cohen's *d* and accompanying 95% confidence interval using a web‐based effect‐size calculator (http://www.campbellcollaboration.org/escalc/html/EffectSizeCalculator-Home.php, Lipsey and Wilson 2001) based on the *P*‐values and sample sizes for these analyses.

#### Gene‐based analyses

On the level of individual dopamine genes, self‐contained tests were conducted in a similar way as the gene‐set analyses, testing the null hypothesis that each individual dopamine gene is not associated with externalizing behavior. Since the gene‐based self‐contained tests were conducted for 12 genes, the significance threshold was adjusted for multiple testing. On the basis of the assumption that genetic distance and the interdependence of genes are correlated with the physical distance between genes (Morton 1955; Pritchard and Przeworski 2001), we calculated the number of independent genes that were separated by at least 5 Mb. According to this calculation the dopamine gene‐set consisted of six independent genes, resulting in a significance threshold of 0.05/6 = 0.0083.

#### SNP level analyses

In addition to our main analyses on gene‐set and gene levels, we ran individual linear regression analyses for all SNPs using PLINK. The previously mentioned covariates, main effects of SNP genotype (additive effect model) and harsh parenting, and the interaction between the SNP and harsh parenting were included in the regression models. Thresholds for significance were adjusted for multiple testing based on the independent number of SNPs, determined by linkage disequilibrium (LD) based pruning in PLINK (−indep‐pairwise 50 5 0.20) of the 151 dopamine SNPs for the total groups of children, for paternal and maternal harsh parenting. LD‐based pruning resulted in 49 independent SNPs, therefore significance thresholds for the analyses at the SNP level were set to *α *= 0.05/49 = 0.0010.

### Additional analysis: are results specific for harsh parenting?

Lastly, we examined whether the gene‐set results were specific for harsh parenting or were accounted for by parental educational level, which differed for the maternal harsh parenting groups, see Table 1.

## Results

Allele frequencies of the individual SNPs did not differ between groups with and without harsh parenting experiences, indicating that the SNPs were not associated with harsh parenting, see Appendix S4. This provides evidence against gene‐environment correlations of child dopamine SNPs and harsh parenting.

### Gene‐set analyses

#### Self‐contained tests

The *P‐*values for the self‐contained gene‐set analyses are presented in Table 3 (Manhattan plots and tables presenting single variant associations with externalizing behavior, used for the calculation of the test statistic, are presented in Appendices S5 and S6. For plots of the test‐statistic distributions of the original data and the permutations see Appendix S7). For paternal harsh parenting, variation in the dopamine gene‐set and externalizing behavior were significantly associated for the children in the group without harsh parenting experiences (*P *=* *0.03). For maternal harsh parenting, this association approached significance in the group without harsh parenting (*P *=* *0.05). The association between dopamine gene‐set variation and externalizing behavior was far from significant in the group with harsh parenting (*P *=* *0.95–0.97).

**Table 3:** *P*‐values for self‐contained tests of associations between dopamine gene‐set or individual dopamine genes and externalizing behavior

	Paternal harsh parenting									Maternal harsh parenting								
Group	Total			Without harsh parenting			With harsh parenting			Total			Without harsh parenting			With harsh parenting		
*n*	1710			1113			597			1881			1208			673		
	*P*	*d*	95% CI *d*	*P*	*d*	95% CI *d*	*P*	*d*	95% CI *d*	*P*	*d*	95% CI *d*	*P*	*d*	95% CI *d*	*P*	*d*	95% CI *d*
Gene‐set analyses:	0.21	0.06	[−0.03, 0.16]	0.03*^A^	0.13	[0.01, 0.25]	0.97	0.00	[−0.16, 0.16]	0.19	0.06	[−0.03, 0.15]	0.05	0.11	[0.00, 0.23]	0.95	0.00	[−0.15, 0.16]
Gene‐based analyses:																		
COMT	0.10	0.08	[−0.02, 0.17]	0.37	0.05	[−0.06, 0.17]	0.37	0.07	[−0.09, 0.23]	0.21	0.06	[−0.03, 0.15]	0.53	0.04	[−0.08, 0.15]	0.23	0.09	[−0.06, 0.24]
DBH	0.96	0.00	[−0.09, 0.10]	0.92	0.01	[−0.11, 0.12]	0.72	0.03	[−0.13, 0.19]	0.92	0.00	[−0.09, 0.10]	0.99	0.00	[−0.11, 0.11]	0.79	0.02	[−0.13, 0.17]
DDC	0.33	0.05	[−0.05, 0.14]	0.11	0.10	[−0.02, 0.21]	0.98	0.00	[−0.16, 0.16]	0.25	0.05	[−0.04, 0.14]	0.16	0.08	[−0.03, 0.19]	0.88	0.01	[−0.14, 0.16]
DRD1	0.51	0.03	[−0.06, 0.13]	0.10	0.10	[−0.02, 0.22]	0.97	0.00	[−0.16, 0.16]	0.33	0.04	[−0.05, 0.14]	0.24	0.07	[−0.05, 0.18]	0.12	0.12	[−0.03, 0.27]
DRD2	0.09	0.08	[−0.01, 0.18]	0.33	0.06	[−0.06, 0.18]	0.32	0.08	[−0.08, 0.24]	0.14	0.07	[−0.02, 0.16]	0.00*^C^	0.17	[0.05, 0.28]	0.92	0.01	[−0.14, 0.16]
DRD3	0.30	0.05	[−0.04, 0.15]	0.24	0.07	[−0.05, 0.19]	0.86	0.01	[−0.15, 0.17]	0.18	0.06	[−0.03, 0.15]	0.50	0.04	[−0.07, 0.15]	0.25	0.09	[−0.06, 0.24]
DRD4	0.83	0.01	[−0.08, 0.11]	1.00	0.00	[−0.12, 0.12]	0.83	0.02	[−0.14, 0.18]	0.51	0.03	[−0.06, 0.12]	0.98	0.00	[−0.11, 0.11]	0.23	0.09	[−0.06, 0.24]
DRD5	×	×	×	×	×	×	×	×	×	×	×	×	×	×	×	×	×	×
DAT	0.03	0.11	[0.01, 0.20]	0.11	0.10	[−0.02, 0.21]	0.46	0.06	[−0.10, 0.22]	0.03	0.10	[0.01, 0.19]	0.01	0.15	[0.04, 0.26]	0.38	0.07	[−0.08, 0.22]
VMAT1	0.43	0.04	[−0.06, 0.13]	0.01*^B^	0.15	[0.04, 0.27]	0.35	0.08	[−0.08, 0.24]	0.32	0.05	[−0.04, 0.14]	0.15	0.08	[−0.03, 0.20]	0.55	0.05	[−0.11, 0.20]
VMAT2	0.92	0.00	[−0.09, 0.10]	0.72	0.02	[−0.10, 0.14]	0.99	0.00	[−0.16, 0.16]	0.83	0.01	[−0.08, 0.10]	0.74	0.02	[−0.09, 0.13]	0.98	0.00	[−0.15, 0.15]
TH	0.42	0.04	[−0.06, 0.13]	0.01	0.15	[0.04, 0.27]	0.59	0.04	[−0.12, 0.20]	0.35	0.04	[−0.05, 0.13]	0.14	0.09	[−0.03, 0.20]	0.86	0.01	[−0.14, 0.16]
DARPP‐32	×	×	×	×	×	×	×	×	×	×	×	×	×	×	×	×	×	×
ANKK1	0.45	0.04	[−0.06, 0.13]	0.68	0.02	[−0.09, 0.14]	0.40	0.07	[−0.09, 0.23]	0.80	0.01	[−0.08, 0.10]	0.52	0.04	[−0.08, 0.15]	0.89	0.01	[−0.14, 0.16]

For the gene‐set, *P*‐values <0.05 are considered significant, for the genes, *P‐*values <0.0083 are considered significant.

*A: *P *= 0.0250, *B: *P *= 0.0068, *C*: P = *0.0040. All other *P*‐values were not significant.

Finally, the self‐contained tests for the total group (groups with and without harsh parenting together) were not significant either (*P *=* *0.19–21). These results seem to indicate that variation in the dopamine gene‐set significantly predicts variance in children's externalizing behavior for children who did not experience harsh parenting, but not for children with harsh parenting experiences. However, given the polygenic nature of the trait, a significant self‐contained *P*‐value may simply reflect this polygenic nature. Therefore competitive tests are run.

#### Competitive tests

Since the self‐contained *P*‐value for the gene‐set analysis in the group without harsh parenting for paternal harsh parenting was significant, a competitive test was conducted. The self‐contained *P*‐value for the dopamine gene‐set (0.03) was compared to the self‐contained *P*‐values for 150 random gene‐sets. The competitive *P*‐value was 0.03, indicating that genetic variation in the dopamine gene‐set was more strongly related to externalizing behavior than genetic variation in random gene‐sets.

#### Interaction of harsh parenting and the dopamine gene‐set

Using a meta‐analysis tool in R (R version 3.1.2, package “metafor”), we compared the *P*‐values for the self‐contained tests of the association between gene‐set variance and externalizing behavior in the groups with and without harsh parenting, as an indirect test of the interaction effect. The results of these tests were *Q*_contrast_ (1) = 0.94 and *P *=* *0.19; *Q*_contrast_ (1) = 0.88, *P *=* *0.28 for paternal and maternal harsh parenting, respectively – indicating that the associations between externalizing behavior and variance in the dopamine gene‐set for the groups with and without harsh parenting were not significantly different, and the interaction was thus not statistically significant.

Additionally, we computed effect estimates (Cohen's *d* and accompanying 95% confidence intervals), based on the *P*‐values and sample sizes for these gene‐set analyses. All *d*'s and accompanying 95% confidence intervals are included in Table 3. For paternal harsh parenting, the effect estimates for the gene‐set analyses were *d* = 0.13 [CI 0.01, 0.25] and *d* = 0.00 [CI −0.16, 0.16] for the groups without and with harsh parenting experiences, respectively. For maternal harsh parenting the effect estimates for the gene‐set analyses were *d* = 0.11 [CI 0.00, 0.23] and *d* = 0.00 [CI −0.15, 0.16] for the groups without and with harsh parenting experiences, respectively.

### Gene‐based analyses

In the gene‐based analyses, associations of the individual dopamine genes with children's externalizing behavior were tested. In Table 3, the *P*‐values for these self‐contained tests are presented; *P*‐values below 0.0083 are considered significant. For paternal harsh parenting, variance in the VMAT1 gene (*P *=* *0.0068) was significantly associated with externalizing behavior, again only in the group without harsh parenting. For maternal harsh parenting, genetic variation in the DRD2 gene was significantly associated with externalizing behavior in the group without harsh parenting (*P *=* *0.0040) but not in the group with harsh parenting or the total group.

#### Interaction of harsh parenting and dopamine genes

We found two significant associations between externalizing behavior and dopamine genes in the group without harsh parenting experiences. For paternal harsh parenting this association was significant for the VMAT1 gene, for maternal harsh parenting the association was significant for the DRD2 gene. We compared the effect sizes based on the *P‐*values for the groups with and without harsh parenting, to test the interactions indirectly. The results of these tests were *Q*_contrast_ (1) = 0.34, *P *=* *0.10 for the VMAT1 gene and *Q*_contrast_ (1) = 0.91, *P *=* *0.08 for the DRD2 gene indicating that the interactions between harsh parenting and variance in the dopamine genes were not statistically significant.

### SNP level analyses

Results of the interaction tests at SNP level are presented in more detail in the Supporting information, (Appendices S5 and S8). At the level of individual SNPs, significant interaction effects were found between paternal harsh parenting and two SNPs: rs1497023 (*β *= 0.92, *P *=* *0.0008) and rs4922132 (*β *= 0.92, *P *=* *0.001). Both SNPs are located on the VMAT1 gene. For maternal harsh parenting, no significant G × E interactions were found at the SNP level.

### Additional analysis: are results specific for harsh parenting?

Finally, we tested whether the results found for harsh parenting were accounted for by the lower parental educational level in the harsh parenting group (see Appendix S9). Results showed that the pattern of results could not be ascribed to differences in educational level.

### Pooled paternal and maternal harsh parenting

In addition to separate analyses for paternal and maternal harsh parenting, we also conducted analyses for the stratified groups based on a pooled harsh parenting measure. For the pooled maternal and paternal harsh parenting, we grouped children into the group with harsh parenting if at least one parent reported harsh parenting behavior, otherwise they were assigned to the group without harsh parenting. None of the self‐contained gene‐set analyses were significant, but the pattern of results was similar, that is, the association between the dopamine gene‐set approached significance for the group without harsh parenting (*P* = 0.08), while it was far from significant in the group with harsh parenting (*P* = 0.92).

## Discussion

In this longitudinal cohort study, we investigated the interplay of harsh parenting and genetic variation across a set of functionally related dopamine genes, in association with children's externalizing behavior. The pattern of effects suggests that the association between genetic variance in the dopamine gene‐set and externalizing behavior depends on harsh parenting experiences: the association between genetic variance in the dopamine gene‐set and externalizing behavior was present for children who did not experience harsh parenting and absent for children who did experience harsh parenting. Results were similar for paternal and maternal harsh parenting. However, the indirect interaction test indicated that the *difference* between the effects for the two harsh parenting groups did not reach significance. Thus, we urge caution in interpreting these results until further replication.

The stratified analyses suggest that unfavorable environmental factors do play a role in explaining the variance in child behavioral outcomes, and that in high‐risk conditions environmental factors may overrule genetic factors. In contrast, low‐risk environments present less environmental “pressure” on the behavioral phenotype, and therefore greater contributions of underlying genetic factors can be expected. In this study, this suggests that the variance in children's externalizing behavior is not explained by underlying genetic variation in the dopamine gene‐set in children who experience harsh parenting. It is important to note that at this level of analysis, we cannot infer the *direction* of effects between genotypes and externalizing behavior. In other words, our analyses do not indicate which genetic variants (alleles) confer greater or lesser risk for externalizing behavior, as they aggregate a combined statistical effect for all SNPs in the gene‐set. This might be possible at the level of individual SNPs –but here we should be cautious due to a lack of conclusive functional knowledge for most SNPs. We can, however, conclude that the genetic variance in a dopamine gene‐set in children in a low‐risk environment explains a significant proportion of the variance in their externalizing behavior. Allele frequencies of the individual genetic variants did not differ between the groups with and without harsh parenting experiences, indicating that there were no evocative gene‐environment correlations between child dopamine SNPs and harsh parenting.

The analyses for single dopamine genes (Table 3) were consistent with the gene‐set findings; significant associations between variance in several single genes were found for the group without harsh parenting. For paternal harsh parenting this association was significant for the VMAT1 gene, for maternal harsh parenting the association was significant for the DRD2 gene. No significant associations between single dopamine genes and externalizing behavior were found for the groups with harsh parenting experiences. At the level of single SNPs, significant interaction effects were found between paternal harsh parenting and two SNPs in VMAT1. In the gene‐set analyses, genetic variation across a large number of SNPs over multiple dopamine genes is aggregated. As a result, SNPs with individual small effects can have a significant combined effect at the level of whole genes or gene‐sets (Winham and Biernacka 2013). This is supported by our results, which are suggestive of G × E effects at the gene‐set level, while at the SNP level, significant interaction effects were found between paternal harsh parenting and only two single SNPs (after correction for multiple testing). Thus, our findings indicate the additional value of including whole gene‐sets as alternative or complement to candidate genes and single SNP analyses that might not reach significance when tested on a one‐by‐one basis.

The groups with and without harsh parenting differed on a number of variables, including psychopathology symptoms of the parents (anxiety and depression), age of the mother, maternal and paternal education level, child externalizing behavior, gender and birth weight of the child and parity. This could mean that the harsh parenting variable might not have been the only pertinent environmental influence. Overall, the group without harsh parenting might be considered a “lower‐risk group”, whereas the group with harsh parenting experiences could be seen as a group exposed to relatively higher risk. Nonetheless, our sample is a population sample and therefore not representative of a true high‐risk group. Our focus on harsh parenting as an environmental factor stemmed from the assumption that parenting is a proximal variable that could mediate the effects of more distal environmental influences such as SES on child behavioral outcomes. Additional analyses showed that the results were not determined by differences in SES. However, based on the current analyses and due to limitations in statistical options it is impossible to fully disentangle the effects of environmental influences and to conclude that the differences between the two groups are only due to the presence or absence of harsh parenting experiences. In future work the gene‐set component of the G × E equation might be complemented by an environment‐set component to account for the potential multifactorial influences of the environment. This approach parallels the search for GWAS x EWAS interactions applied to type 2 diabetes mellitus (Patel et al. 2013) which throws a larger net on the potential pertinent environment and at the same time compensates for multiple testing involved in scrutinizing gene‐set x environment‐set interactions.

A few issues are important to consider when interpreting our results. First, significant self‐contained *P*‐values for polygenic traits could be caused by this polygenic background. Therefore, competitive tests are needed to see whether the gene‐set of interest is more strongly associated with the outcome than randomly generated matched gene‐sets. We conducted competitive tests whenever the self‐contained test reached significance. This was only the case for paternal harsh parenting, in the group without harsh parenting experiences. The competitive test also proved to be significant for this group, indicating that the association between externalizing behavior and the dopamine gene‐set was stronger than it was for 150 random gene‐sets. In the group with paternal harsh parenting experiences, the self‐contained test was far from significant. Similarly, for maternal harsh parenting, none of the self‐contained tests reached significance and thus competitive tests were not conducted. Although these results do not allow firm conclusions, it should be noted that the directions of the effects were similar to those of paternal harsh parenting.

A second issue to keep in mind when interpreting our results is multiple testing. We performed multiple tests at the levels of single genes and SNPs in groups based on three related harsh parenting variables. Since the genes and SNPs were not completely independent, we judged it inappropriate to use a highly stringent Bonferroni correction in this first exploratory study of its kind. More conservative approaches might be taken in future replication efforts.

A third issue to consider are sample size differences between the groups with and without harsh parenting. The lack of an association in the groups with harsh parenting experiences might be alternatively explained by smaller sample size. However, this is not reflected in the effect sizes for the gene‐set analyses, based on the *P*‐values and sample sizes.

This study has a number of notable strengths. First, our study sample is a large ethnically homogenous subsample from a longitudinal population‐based cohort study. Secondly, we had available data on harsh parenting in both fathers and mothers from the same families. Our study has some limitations as well. Harsh parenting was assessed by self‐report, which might be open to bias (e.g., Morsbach and Prinz 2006). Valid assessment of the environment is shown to be critical for G × E results. Therefore, it is preferable to assess environmental factors by observational measures or interviews in future studies (Uher and McGuffin 2010; McGuffin et al. 2011). Second, both parent and child behavior was assessed through questionnaires filled out by the parents, which could have resulted in reporter bias. A third limitation is that the specifications of the JAG tool implied restriction to a dichotomous rather than continuous environmental measure. The harsh parenting group thus represents a group with harsh parenting ranging from moderate to extreme. Note, however, that this may partly tackle the limitation of self‐report: Parents who did not completely deny but underreported their level of harsh parenting were classified in the group with harsh parenting. Another limitation could be that the selection of the dopamine genes in the gene‐set is not exclusively implicated in the dopamine system and is not the only selection possible, and a larger variety of genes might be included in future studies. Future research may also include other biological gene‐sets. Finally, even though our sample was of considerable size for gene‐set analyses, larger samples might improve power, particularly when splitting the sample to investigate G × E effects. Replication of our exploratory findings is needed.

In sum, we found suggestive evidence for interplay between harsh parenting and dopamine genetic variance, with an association between dopamine genes and externalizing behavior for children who did not experience harsh parenting. Harsh parenting may overrule the role of genetic factors in externalizing behavior. The approach presented here, involving gene and gene‐set analyses, offers a useful and relatively underexplored alternative to studies focusing on single candidate polymorphisms in the search for genetic variation underlying complex behavior.

## Conflict of Interest

F. C. Verhulst publishes the Dutch versions of the ASEBA from which he receives remuneration.

## Supporting information


**Appendix S1.** Genotyping of DRD4 48 bp VNTR
**Appendix S2.** Minor Allele Frequencies of genetic variants across groups.
**Appendix S3.** Harsh parenting groupings measures: distribution of sum scores, and information on dichotomization.
**Appendix S4.** Associations of individual genetic variants and harsh parenting paternal/maternal (comparison allele frequencies between groups with and without harsh parenting).
**Appendix S5.** Manhattan plots.
**Appendix S6.** Tables with p‐values for single SNP associations with externalizing behavior (TEST = ADD), from gene‐set analyses – corresponding to Manhattan plots ABC (For paternal/maternal).
**Appendix S7.** Plots of test‐statistic distributions of the original data and the permutations of the self‐contained gene‐set analyses in JAG.
**Appendix S8.** Tests of the interaction effect of SNP and harsh parenting (TEST = ADD X Harsh Parenting) for the total group (corresponding to Manhattan plots D).
**Appendix S9.** Additional analyses: gene‐set analyses with educational level.
