# Supplementary material for: Beyond main effects of gene‐sets: harsh parenting moderates the association between a dopamine gene‐set and child externalizing behavior
Source: Brain Behav. 2016 May 31;6(8):e00498. doi: 10.1002/brb3.498 (PMC4980469; doi:10.1002/brb3.498)
Supplement: Supplementary file 1 — Appendix S1. Genotyping of DRD4 48 bp VNTR Appendix S2. Minor Allele Frequencies of genetic variants across groups. Appendix S3. Harsh parenting groupings measures: distribution of sum scores, and information on dichotomization. Appendix S4. Associations of individual genetic variants and harsh parenting paternal/maternal (comparison allele frequencies between groups with and without harsh parenting). Appendix S5. Manhattan plots. Appendix S6. Tables with p‐values for single SNP associations with externalizing behavior (TEST = ADD), from gene‐set analyses – corresponding to Manhattan plots ABC (For paternal/maternal). Appendix S7. Plots of test‐statistic distributions of the original data and the permutations of the self‐contained gene‐set analyses in JAG. Appendix S8. Tests of the interaction effect of SNP and harsh parenting (TEST = ADD X Harsh Parenting) for the total group (corresponding to Manhattan plots D). Appendix S9. Additional analyses: gene‐set analyses with educational level. [file BRB3-6-e00498-s001.docx]

**Appendices Manuscript: Beyond main effects of gene-sets. Harsh parenting moderates the association between a dopamine gene-set and child externalizing behavior.**

D.A. Windhorst, V.R. Mileva-Seitz, R.C.A. Rippe, H. Tiemeier, V.W.V. Jaddoe, F.C. Verhulst, M.H. van IJzendoorn, & M.J. Bakermans-Kranenburg

**Content:**

| S1. | Genotyping of DRD4 48 bp VNTR | p. 2 |
| --- | --- | --- |
| S2. | Minor Allele Frequencies of genetic variants across groups | p. 3 |
| S3. | Harsh parenting groupings measures: distribution of sum scores, and information on dichotomization. | p. 8 |
| S4. | Associations of individual genetic variants and harsh parenting paternal/maternal (comparison allele-frequencies between groups with and without harsh parenting) | p. 10 |
| S5. | Manhattan plots | p. 15 |
| S6. | Tables with p-values for single SNP associations with externalizing behavior (TEST = ADD), from gene-set analyses – corresponding to Manhattan plots ABC (For paternal/maternal). | p.17 |
| S7. | Plots of test-statistic distributions of the original data and the permutations of the self-contained gene-set analyses in JAG. | p. 21 |
| S8. | Tests of the interaction effect of SNP and harsh parenting (TEST = ADD X Harsh Parenting) for the total group (corresponding to Manhattan plots D) | p. 24 |
| S9. | Additional analyses: gene-set analyses with educational level | p. 29 |

***S1. Genotyping of DRD4 48 bp VNTR***

DNA was collected from cord blood samples at birth. Genotyping of the DRD4 48 bp VNTR was amplified using primers D4-FGCGACTACGTGGTCTACTCG and D4-R-AGGACCCTCATGGCCTTG. Reactions were performed in a 384-wells format in a total reaction volume of 10 ul containing 10 ng DNA, 1 pmol/ul of each primer, 0,4 mM dNTPs, 1 M betaine, 1x GC buffer I (Takara Bio Inc., Otsu, Japan) and 0,5 U/ul LA Taq (Takara Bio Inc.). PCR cycling consisted of initial denaturation of 1 min at 94° C, and 34 cycles with denaturation of 30 seconds at 95°C, annealing of 30 seconds at 58°C and extension of 1 minute at 71 72°C. PCR fragments were size-separated on the Labchip GX (Caliper Life Sciences, Hopkinton, MA) using a HT DNA 5K chip (Caliper Life sciences). The number of DRD4 repeats was determined using the size of the PCR-fragments. To assure genotyping accuracy 225 (18%) random samples were genotyped for a second time. Virtually all samples (98.7%) gave the same genotypes.
To match the bi-allelic format of the genotyped SNPs, DRD4 VNTR genotype was recoded as 0, 1, or 2, based on the number of 7-repeat alleles present. HWE *p-*value was 0.05, MAF was 0.20. The DRD4 VNTR was missing for 10.4 % of the Caucasian children with GWAS data (available for n = 2537).

***S2. Minor Allele Frequencies of genetic variants across groups***

|  |  |  |  | **Paternal** | | |  | **Maternal** | | |
| --- | --- | --- | --- | --- | --- | --- | --- | --- | --- | --- |
| **SNP** | **gene** | **gene** | **CHR** | **Total** | **Without harsh parenting** | **With**  **harsh parenting** |  | **Total** | **Without**  **harsh parenting** | **With**  **harsh parenting** |
| RS1544325 | 1312 | COMT | 22 | 0.4704 | 0.4681 | 0.4748 |  | 0.4731 | 0.4764 | 0.4673 |
| RS165599 | 1312 | COMT | 22 | 0.2881 | 0.2943 | 0.2764 |  | 0.2855 | 0.2952 | 0.2682 |
| RS165656 | 1312 | COMT | 22 | 0.4561 | 0.4663 | 0.4371 |  | 0.4574 | 0.4561 | 0.4598 |
| RS165722 | 1312 | COMT | 22 | 0.4577 | 0.4670 | 0.4403 |  | 0.4589 | 0.4576 | 0.4613 |
| RS165728 | 1312 | COMT | 22 | 0.0471 | 0.0449 | 0.0511 |  | 0.0457 | 0.0422 | 0.0520 |
| RS165774 | 1312 | COMT | 22 | 0.3186 | 0.3111 | 0.3325 |  | 0.3164 | 0.3172 | 0.3149 |
| RS174675 | 1312 | COMT | 22 | 0.2670 | 0.2680 | 0.2652 |  | 0.2640 | 0.2527 | 0.2842 |
| RS174699 | 1312 | COMT | 22 | 0.0477 | 0.0445 | 0.0536 |  | 0.0463 | 0.0426 | 0.0528 |
| RS2239393 | 1312 | COMT | 22 | 0.3847 | 0.3948 | 0.3660 |  | 0.3886 | 0.3898 | 0.3863 |
| RS4646312 | 1312 | COMT | 22 | 0.3823 | 0.3933 | 0.3618 |  | 0.3853 | 0.3868 | 0.3826 |
| RS4646316 | 1312 | COMT | 22 | 0.2392 | 0.2399 | 0.2379 |  | 0.2414 | 0.2318 | 0.2585 |
| RS4680 | 1312 | COMT | 22 | 0.4582 | 0.4672 | 0.4413 |  | 0.4593 | 0.4582 | 0.4613 |
| RS5993883 | 1312 | COMT | 22 | 0.4909 | 0.4910 | 0.4908 |  | 0.4888 | 0.4934 | 0.4807 |
| RS737866 | 1312 | COMT | 22 | 0.2648 | 0.2657 | 0.2630 |  | 0.2649 | 0.2726 | 0.2511 |
| RS10993949 | 1621 | DBH | 9 | 0.0094 | 0.0090 | 0.0101 |  | 0.0090 | 0.0099 | 0.0075 |
| RS1108581 | 1621 | DBH | 9 | 0.2012 | 0.2017 | 0.2002 |  | 0.1962 | 0.1999 | 0.1895 |
| RS1541332 | 1621 | DBH | 9 | 0.4497 | 0.4492 | 0.4506 |  | 0.4482 | 0.4466 | 0.4510 |
| RS1611123 | 1621 | DBH | 9 | 0.4725 | 0.4730 | 0.4715 |  | 0.4724 | 0.4694 | 0.4777 |
| RS2007153 | 1621 | DBH | 9 | 0.3635 | 0.3661 | 0.3585 |  | 0.3591 | 0.3605 | 0.3566 |
| RS2097628 | 1621 | DBH | 9 | 0.3882 | 0.3998 | 0.3666 |  | 0.3881 | 0.3821 | 0.3991 |
| RS2283123 | 1621 | DBH | 9 | 0.1147 | 0.1235 | 0.0982 |  | 0.1146 | 0.1138 | 0.1161 |
| RS2283124 | 1621 | DBH | 9 | 0.1146 | 0.1235 | 0.0980 |  | 0.1146 | 0.1138 | 0.1159 |
| RS2519143 | 1621 | DBH | 9 | 0.1917 | 0.1920 | 0.1913 |  | 0.1927 | 0.1950 | 0.1885 |
| RS2519154 | 1621 | DBH | 9 | 0.4225 | 0.4227 | 0.4221 |  | 0.4261 | 0.4247 | 0.4287 |
| RS2519155 | 1621 | DBH | 9 | 0.3485 | 0.3477 | 0.3501 |  | 0.3527 | 0.3498 | 0.3581 |
| RS2797853 | 1621 | DBH | 9 | 0.3424 | 0.3441 | 0.3392 |  | 0.3461 | 0.3448 | 0.3484 |
| RS2873804 | 1621 | DBH | 9 | 0.4623 | 0.4605 | 0.4657 |  | 0.4628 | 0.4619 | 0.4643 |
| RS3025382 | 1621 | DBH | 9 | 0.1157 | 0.1166 | 0.1141 |  | 0.1145 | 0.1156 | 0.1125 |
| RS3025388 | 1621 | DBH | 9 | 0.1621 | 0.1649 | 0.1569 |  | 0.1566 | 0.1573 | 0.1555 |
| RS5320 | 1621 | DBH | 9 | 0.0526 | 0.0530 | 0.0519 |  | 0.0492 | 0.0472 | 0.0528 |
| RS77905 | 1621 | DBH | 9 | 0.4743 | 0.4726 | 0.4774 |  | 0.4795 | 0.4839 | 0.4717 |
| RS10268819 | 1644 | DDC | 7 | 0.1175 | 0.1177 | 0.1173 |  | 0.1162 | 0.1147 | 0.1189 |
| RS10499694 | 1644 | DDC | 7 | 0.4883 | 0.4847 | 0.4950 |  | 0.4867 | 0.4930 | 0.4754 |
| RS10499695 | 1644 | DDC | 7 | 0.4868 | 0.4811 | 0.4975 |  | 0.4864 | 0.4917 | 0.4769 |
| RS10499696 | 1644 | DDC | 7 | 0.1234 | 0.1249 | 0.1206 |  | 0.1217 | 0.1175 | 0.1293 |
| RS11238131 | 1644 | DDC | 7 | 0.2946 | 0.2929 | 0.2978 |  | 0.2920 | 0.2926 | 0.2909 |
| RS11238133 | 1644 | DDC | 7 | 0.3405 | 0.3353 | 0.3501 |  | 0.3369 | 0.3396 | 0.3321 |
| RS11575286 | 1644 | DDC | 7 | 0.1080 | 0.1075 | 0.1089 |  | 0.1104 | 0.1126 | 0.1064 |
| RS11575387 | 1644 | DDC | 7 | 0.0702 | 0.0719 | 0.0670 |  | 0.0726 | 0.0700 | 0.0773 |
| RS11575489 | 1644 | DDC | 7 | 0.0170 | 0.0157 | 0.0193 |  | 0.0186 | 0.0199 | 0.0163 |
| RS11575522 | 1644 | DDC | 7 | 0.0184 | 0.0193 | 0.0168 |  | 0.0186 | 0.0195 | 0.0171 |
| RS11575542 | 1644 | DDC | 7 | 0.0159 | 0.0167 | 0.0143 |  | 0.0163 | 0.0175 | 0.0142 |
| RS11768267 | 1644 | DDC | 7 | 0.4392 | 0.4380 | 0.4414 |  | 0.4383 | 0.4408 | 0.4339 |
| RS1349492 | 1644 | DDC | 7 | 0.4205 | 0.4182 | 0.4246 |  | 0.4197 | 0.4201 | 0.4190 |
| RS1376523 | 1644 | DDC | 7 | 0.0269 | 0.0261 | 0.0285 |  | 0.0292 | 0.0298 | 0.0282 |
| RS17133877 | 1644 | DDC | 7 | 0.0383 | 0.0377 | 0.0394 |  | 0.0401 | 0.0422 | 0.0364 |
| RS17634958 | 1644 | DDC | 7 | 0.1275 | 0.1276 | 0.1273 |  | 0.1287 | 0.1304 | 0.1257 |
| RS1966839 | 1644 | DDC | 7 | 0.3477 | 0.3428 | 0.3568 |  | 0.3480 | 0.3551 | 0.3351 |
| RS2329340 | 1644 | DDC | 7 | 0.3450 | 0.3392 | 0.3559 |  | 0.3445 | 0.3518 | 0.3314 |
| RS2329371 | 1644 | DDC | 7 | 0.2146 | 0.2115 | 0.2203 |  | 0.2105 | 0.2146 | 0.2031 |
| RS3735274 | 1644 | DDC | 7 | 0.2453 | 0.2435 | 0.2487 |  | 0.2477 | 0.2492 | 0.2452 |
| RS3779084 | 1644 | DDC | 7 | 0.2060 | 0.2089 | 0.2005 |  | 0.2104 | 0.2096 | 0.2117 |
| RS3807552 | 1644 | DDC | 7 | 0.2331 | 0.2308 | 0.2374 |  | 0.2281 | 0.2330 | 0.2193 |
| RS3807553 | 1644 | DDC | 7 | 0.1082 | 0.1078 | 0.1089 |  | 0.1106 | 0.1126 | 0.1070 |
| RS3807558 | 1644 | DDC | 7 | 0.2156 | 0.2107 | 0.2247 |  | 0.2109 | 0.2125 | 0.2081 |
| RS3829897 | 1644 | DDC | 7 | 0.3898 | 0.3850 | 0.3987 |  | 0.3921 | 0.4023 | 0.3737 |
| RS4947631 | 1644 | DDC | 7 | 0.0190 | 0.0175 | 0.0218 |  | 0.0205 | 0.0211 | 0.0193 |
| RS6592952 | 1644 | DDC | 7 | 0.4211 | 0.4187 | 0.4255 |  | 0.4203 | 0.4209 | 0.4190 |
| RS6592961 | 1644 | DDC | 7 | 0.2156 | 0.2181 | 0.2111 |  | 0.2198 | 0.2204 | 0.2188 |
| RS6593011 | 1644 | DDC | 7 | 0.1529 | 0.1527 | 0.1533 |  | 0.1518 | 0.1498 | 0.1553 |
| RS732215 | 1644 | DDC | 7 | 0.4468 | 0.4485 | 0.4436 |  | 0.4484 | 0.4480 | 0.4491 |
| RS7809758 | 1644 | DDC | 7 | 0.3500 | 0.3527 | 0.3451 |  | 0.3549 | 0.3539 | 0.3566 |
| RS880028 | 1644 | DDC | 7 | 0.2058 | 0.2084 | 0.2010 |  | 0.2103 | 0.2094 | 0.2117 |
| RS5326 | 1812 | DRD1 | 5 | 0.1632 | 0.1577 | 0.1734 |  | 0.1637 | 0.1697 | 0.1530 |
| RS686 | 1812 | DRD1 | 5 | 0.3810 | 0.3823 | 0.3786 |  | 0.3796 | 0.3738 | 0.3900 |
| RS1076563 | 1813 | DRD2 | 11 | 0.3804 | 0.3827 | 0.3760 |  | 0.3817 | 0.3833 | 0.3789 |
| RS1079727 | 1813 | DRD2 | 11 | 0.1395 | 0.1375 | 0.1432 |  | 0.1377 | 0.1370 | 0.1389 |
| RS11214606 | 1813 | DRD2 | 11 | 0.0565 | 0.0580 | 0.0536 |  | 0.0545 | 0.0543 | 0.0550 |
| RS17529477 | 1813 | DRD2 | 11 | 0.3307 | 0.3311 | 0.3300 |  | 0.3349 | 0.3332 | 0.3380 |
| RS2440390 | 1813 | DRD2 | 11 | 0.1368 | 0.1384 | 0.1340 |  | 0.1398 | 0.1411 | 0.1374 |
| RS2471857 | 1813 | DRD2 | 11 | 0.1383 | 0.1366 | 0.1415 |  | 0.1364 | 0.1362 | 0.1367 |
| RS2734838 | 1813 | DRD2 | 11 | 0.3801 | 0.3827 | 0.3752 |  | 0.3814 | 0.3833 | 0.3782 |
| RS4274224 | 1813 | DRD2 | 11 | 0.4912 | 0.4933 | 0.4874 |  | 0.4928 | 0.4892 | 0.4993 |
| RS4620755 | 1813 | DRD2 | 11 | 0.1070 | 0.1101 | 0.1013 |  | 0.1066 | 0.1080 | 0.1040 |
| RS4648317 | 1813 | DRD2 | 11 | 0.1398 | 0.1366 | 0.1460 |  | 0.1410 | 0.1411 | 0.1406 |
| RS4648318 | 1813 | DRD2 | 11 | 0.2415 | 0.2421 | 0.2404 |  | 0.2448 | 0.2475 | 0.2400 |
| RS4648319 | 1813 | DRD2 | 11 | 0.1425 | 0.1398 | 0.1474 |  | 0.1402 | 0.1400 | 0.1406 |
| RS4938019 | 1813 | DRD2 | 11 | 0.1401 | 0.1379 | 0.1441 |  | 0.1406 | 0.1411 | 0.1397 |
| RS7125415 | 1813 | DRD2 | 11 | 0.0799 | 0.0823 | 0.0754 |  | 0.0801 | 0.0816 | 0.0773 |
| RS7131056 | 1813 | DRD2 | 11 | 0.4167 | 0.4203 | 0.4101 |  | 0.4144 | 0.4156 | 0.4122 |
| RS10934256 | 1814 | DRD3 | 3 | 0.1880 | 0.1855 | 0.1926 |  | 0.1890 | 0.1892 | 0.1887 |
| RS11706283 | 1814 | DRD3 | 3 | 0.0986 | 0.0988 | 0.0982 |  | 0.0978 | 0.0914 | 0.1094 |
| RS1486009 | 1814 | DRD3 | 3 | 0.0591 | 0.0598 | 0.0578 |  | 0.0588 | 0.0571 | 0.0617 |
| RS167770 | 1814 | DRD3 | 3 | 0.2737 | 0.2736 | 0.2739 |  | 0.2735 | 0.2728 | 0.2749 |
| RS2134655 | 1814 | DRD3 | 3 | 0.2561 | 0.2534 | 0.2613 |  | 0.2600 | 0.2674 | 0.2467 |
| RS226082 | 1814 | DRD3 | 3 | 0.2740 | 0.2736 | 0.2747 |  | 0.2741 | 0.2736 | 0.2749 |
| RS2630349 | 1814 | DRD3 | 3 | 0.0626 | 0.0623 | 0.0632 |  | 0.0599 | 0.0586 | 0.0621 |
| RS2630351 | 1814 | DRD3 | 3 | 0.0621 | 0.0617 | 0.0629 |  | 0.0594 | 0.0580 | 0.0618 |
| RS324029 | 1814 | DRD3 | 3 | 0.2743 | 0.2738 | 0.2752 |  | 0.2741 | 0.2736 | 0.2749 |
| RS7633291 | 1814 | DRD3 | 3 | 0.1880 | 0.1855 | 0.1926 |  | 0.1890 | 0.1892 | 0.1887 |
| RS9288993 | 1814 | DRD3 | 3 | 0.0260 | 0.0283 | 0.0218 |  | 0.0258 | 0.0265 | 0.0246 |
| RS963468 | 1814 | DRD3 | 3 | 0.4090 | 0.4137 | 0.4000 |  | 0.4079 | 0.4031 | 0.4165 |
| DRD4R | 1815 | DRD4 | 11 | 0.2033 | 0.1937 | 0.2209 |  | 0.2012 | 0.2032 | 0.1977 |
| RS2617605 | 6531 | DAT | 5 | 0.3611 | 0.3706 | 0.3434 |  | 0.3543 | 0.3638 | 0.3373 |
| RS27048 | 6531 | DAT | 5 | 0.4620 | 0.4717 | 0.4439 |  | 0.4556 | 0.4636 | 0.4413 |
| RS27072 | 6531 | DAT | 5 | 0.1966 | 0.2064 | 0.1784 |  | 0.1923 | 0.1959 | 0.1857 |
| RS3776511 | 6531 | DAT | 5 | 0.1870 | 0.1794 | 0.2010 |  | 0.1912 | 0.1900 | 0.1935 |
| RS3776512 | 6531 | DAT | 5 | 0.1868 | 0.1794 | 0.2005 |  | 0.1911 | 0.1900 | 0.1930 |
| RS40184 | 6531 | DAT | 5 | 0.4722 | 0.4829 | 0.4523 |  | 0.4710 | 0.4739 | 0.4658 |
| RS403636 | 6531 | DAT | 5 | 0.1521 | 0.1497 | 0.1566 |  | 0.1540 | 0.1424 | 0.1749 |
| RS460000 | 6531 | DAT | 5 | 0.1939 | 0.1882 | 0.2044 |  | 0.1935 | 0.1941 | 0.1924 |
| RS460700 | 6531 | DAT | 5 | 0.1934 | 0.1879 | 0.2035 |  | 0.1931 | 0.1939 | 0.1917 |
| RS464049 | 6531 | DAT | 5 | 0.4087 | 0.4074 | 0.4112 |  | 0.4056 | 0.4094 | 0.3988 |
| RS6347 | 6531 | DAT | 5 | 0.2626 | 0.2527 | 0.2810 |  | 0.2650 | 0.2608 | 0.2727 |
| RS6350 | 6531 | DAT | 5 | 0.0521 | 0.0553 | 0.0461 |  | 0.0529 | 0.0555 | 0.0483 |
| RS6869645 | 6531 | DAT | 5 | 0.0708 | 0.0714 | 0.0695 |  | 0.0694 | 0.0687 | 0.0706 |
| RS12545707 | 6570 | VMAT1 | 8 | 0.2654 | 0.2621 | 0.2714 |  | 0.2699 | 0.2626 | 0.2831 |
| RS13258461 | 6570 | VMAT1 | 8 | 0.4707 | 0.4685 | 0.4749 |  | 0.4694 | 0.4760 | 0.4577 |
| RS1390938 | 6570 | VMAT1 | 8 | 0.2633 | 0.2648 | 0.2605 |  | 0.2606 | 0.2734 | 0.2377 |
| RS1390939 | 6570 | VMAT1 | 8 | 0.4439 | 0.4434 | 0.4447 |  | 0.4365 | 0.4429 | 0.4250 |
| RS1497020 | 6570 | VMAT1 | 8 | 0.3226 | 0.3242 | 0.3196 |  | 0.3180 | 0.3342 | 0.2890 |
| RS1497022 | 6570 | VMAT1 | 8 | 0.3854 | 0.3876 | 0.3813 |  | 0.3902 | 0.3867 | 0.3964 |
| RS1497023 | 6570 | VMAT1 | 8 | 0.1764 | 0.1794 | 0.1709 |  | 0.1750 | 0.1864 | 0.1545 |
| RS1497025 | 6570 | VMAT1 | 8 | 0.1755 | 0.1794 | 0.1681 |  | 0.1741 | 0.1864 | 0.1520 |
| RS2270637 | 6570 | VMAT1 | 8 | 0.1852 | 0.1826 | 0.1901 |  | 0.1859 | 0.1823 | 0.1924 |
| RS2270649 | 6570 | VMAT1 | 8 | 0.0026 | 0.0036 | 0.0008 |  | 0.0024 | 0.0025 | 0.0022 |
| RS2270650 | 6570 | VMAT1 | 8 | 0.3711 | 0.3724 | 0.3685 |  | 0.3735 | 0.3742 | 0.3722 |
| RS3779672 | 6570 | VMAT1 | 8 | 0.1623 | 0.1595 | 0.1675 |  | 0.1590 | 0.1519 | 0.1716 |
| RS4921692 | 6570 | VMAT1 | 8 | 0.0882 | 0.0842 | 0.0956 |  | 0.0873 | 0.0815 | 0.0976 |
| RS4922132 | 6570 | VMAT1 | 8 | 0.1716 | 0.1752 | 0.1650 |  | 0.1707 | 0.1838 | 0.1471 |
| RS6586896 | 6570 | VMAT1 | 8 | 0.0936 | 0.0957 | 0.0898 |  | 0.0931 | 0.0960 | 0.0878 |
| RS6586897 | 6570 | VMAT1 | 8 | 0.4304 | 0.4308 | 0.4296 |  | 0.4240 | 0.4305 | 0.4123 |
| RS6992927 | 6570 | VMAT1 | 8 | 0.2642 | 0.2662 | 0.2605 |  | 0.2622 | 0.2751 | 0.2392 |
| RS721950 | 6570 | VMAT1 | 8 | 0.3739 | 0.3747 | 0.3725 |  | 0.3801 | 0.3762 | 0.3869 |
| RS7841346 | 6570 | VMAT1 | 8 | 0.2050 | 0.2013 | 0.2119 |  | 0.2060 | 0.2003 | 0.2162 |
| RS952860 | 6570 | VMAT1 | 8 | 0.0035 | 0.0054 | 0.0000 |  | 0.0032 | 0.0041 | 0.0015 |
| RS10082463 | 6571 | VMAT2 | 10 | 0.0939 | 0.0894 | 0.1022 |  | 0.0936 | 0.1022 | 0.0780 |
| RS11197936 | 6571 | VMAT2 | 10 | 0.3806 | 0.3783 | 0.3849 |  | 0.3838 | 0.3873 | 0.3774 |
| RS1860404 | 6571 | VMAT2 | 10 | 0.1563 | 0.1632 | 0.1435 |  | 0.1551 | 0.1645 | 0.1384 |
| RS2015586 | 6571 | VMAT2 | 10 | 0.4398 | 0.4308 | 0.4564 |  | 0.4378 | 0.4367 | 0.4398 |
| RS2283138 | 6571 | VMAT2 | 10 | 0.1080 | 0.1065 | 0.1107 |  | 0.1077 | 0.1168 | 0.0914 |
| RS3523 | 6571 | VMAT2 | 10 | 0.1465 | 0.1469 | 0.1457 |  | 0.1438 | 0.1469 | 0.1382 |
| RS363221 | 6571 | VMAT2 | 10 | 0.0597 | 0.0621 | 0.0553 |  | 0.0588 | 0.0580 | 0.0603 |
| RS363224 | 6571 | VMAT2 | 10 | 0.4661 | 0.4573 | 0.4824 |  | 0.4636 | 0.4619 | 0.4666 |
| RS363225 | 6571 | VMAT2 | 10 | 0.4658 | 0.4573 | 0.4816 |  | 0.4631 | 0.4599 | 0.4688 |
| RS363227 | 6571 | VMAT2 | 10 | 0.1134 | 0.1104 | 0.1189 |  | 0.1126 | 0.1191 | 0.1010 |
| RS363230 | 6571 | VMAT2 | 10 | 0.4904 | 0.4951 | 0.4816 |  | 0.4952 | 0.4983 | 0.4837 |
| RS363251 | 6571 | VMAT2 | 10 | 0.3735 | 0.3793 | 0.3628 |  | 0.3740 | 0.3816 | 0.3604 |
| RS363275 | 6571 | VMAT2 | 10 | 0.1459 | 0.1460 | 0.1457 |  | 0.1433 | 0.1465 | 0.1374 |
| RS363276 | 6571 | VMAT2 | 10 | 0.1451 | 0.1448 | 0.1457 |  | 0.1426 | 0.1454 | 0.1374 |
| RS363332 | 6571 | VMAT2 | 10 | 0.2648 | 0.2673 | 0.2601 |  | 0.2649 | 0.2724 | 0.2515 |
| RS363341 | 6571 | VMAT2 | 10 | 0.2838 | 0.2866 | 0.2785 |  | 0.2856 | 0.2928 | 0.2727 |
| RS363387 | 6571 | VMAT2 | 10 | 0.0342 | 0.0350 | 0.0327 |  | 0.0346 | 0.0368 | 0.0305 |
| RS363397 | 6571 | VMAT2 | 10 | 0.0354 | 0.0359 | 0.0343 |  | 0.0356 | 0.0377 | 0.0320 |
| RS2070762 | 7054 | TH | 11 | 0.4985 | 0.4951 | 0.4950 |  | 0.4992 | 0.4988 | 0.5000 |
| RS6356 | 7054 | TH | 11 | 0.3548 | 0.3551 | 0.3543 |  | 0.3568 | 0.3592 | 0.3525 |
| RS1800497 | 255239 | ANKK1 | 11 | 0.1887 | 0.1891 | 0.1879 |  | 0.1862 | 0.1860 | 0.1865 |
| RS2734848 | 255239 | ANKK1 | 11 | 0.1998 | 0.2019 | 0.1960 |  | 0.2040 | 0.2125 | 0.1887 |
| RS2734849 | 255239 | ANKK1 | 11 | 0.4889 | 0.4951 | 0.4774 |  | 0.4912 | 0.5000 | 0.4755 |
| RS4590907 | 255239 | ANKK1 | 11 | 0.1266 | 0.1271 | 0.1256 |  | 0.1271 | 0.1267 | 0.1278 |
| RS7118900 | 255239 | ANKK1 | 11 | 0.1767 | 0.1790 | 0.1725 |  | 0.1742 | 0.1748 | 0.1731 |
|  |  |  |  |  |  |  |  |  |  |  |
| MAF < 0.01 |  |  |  |  |  |  |  |  |  |  |
| MAF < 0.001 |  |  |  |  |  |  |  |  |  |  |

***S3. Harsh parenting groupings measures: distribution of sum scores, and information on dichotomization.***

**Paternal Harsh parenting, weighted sum score based on 5 items (yelling excluded)
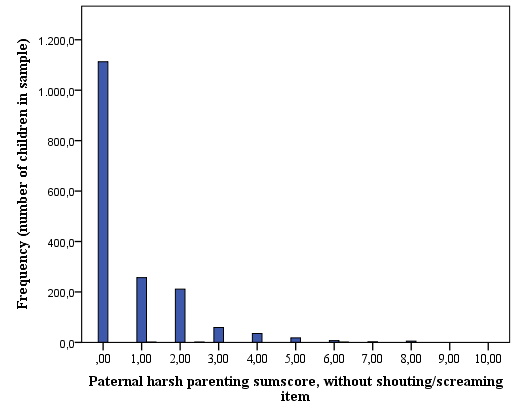
**

A: group without harsh parenting

B: group with harsh parenting

A B

| **Weighted sum score** | | **Frequency** | **Percent** | A: group without harsh parenting  B: group with harsh parenting |
| --- | --- | --- | --- | --- |
|  | ,00 | 1113 | 65,1 | A |
|  | 1,00 | 257 | 15,0 | B |
|  | 1,25 | 1 | ,1 |  |
|  | 2,00 | 211 | 12,3 |  |
|  | 2,50 | 1 | ,1 |  |
|  | 3,00 | 59 | 3,5 |  |
|  | 4,00 | 35 | 2,0 |  |
|  | 5,00 | 18 | 1,1 |  |
|  | 6,00 | 7 | ,4 |  |
|  | 6,25 | 1 | ,1 |  |
|  | 7,00 | 2 | ,1 |  |
|  | 8,00 | 5 | ,3 |  |
|  | Total | 1710 | 100,0 |  |

**Maternal Harsh parenting, weighted sum score based on 5 items (yelling excluded)**


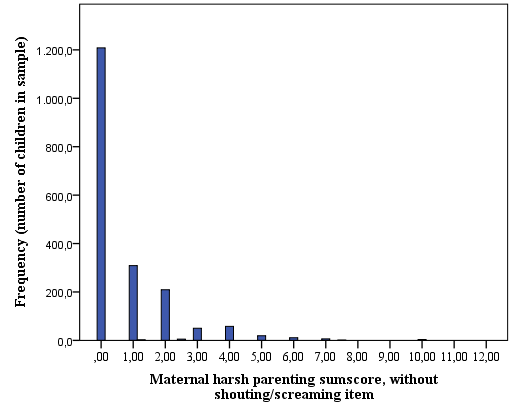


A: group without harsh parenting

B: group with harsh parenting

A B

| **Weighted sum score** | | **Frequency** | **Percent** | A: group without harsh parenting  B: group with harsh parenting |
| --- | --- | --- | --- | --- |
|  | ,00 | 1208 | 64,2 | A |
|  | 1,00 | 309 | 16,4 | B |
|  | 1,25 | 2 | ,1 |  |
|  | 2,00 | 209 | 11,1 |  |
|  | 2,50 | 5 | ,3 |  |
|  | 3,00 | 50 | 2,7 |  |
|  | 4,00 | 58 | 3,1 |  |
|  | 5,00 | 19 | 1,0 |  |
|  | 6,00 | 11 | ,6 |  |
|  | 7,00 | 6 | ,3 |  |
|  | 7,50 | 1 | ,1 |  |
|  | 10,00 | 3 | ,2 |  |
|  | Total | 1881 | 100,0 |  |
|  |  |  |  |  |

***S4. Associations of individual genetic variants and harsh parenting paternal/maternal (comparison allele-frequencies between groups with and without harsh parenting)*** **included covariates: 4 principal components of GWAS data, child age and gender*

|  |  |  |  | **Paternal** | | | |  | **Maternal** | | | |
| --- | --- | --- | --- | --- | --- | --- | --- | --- | --- | --- | --- | --- |
| **SNP** | **Gene** | **Gene** | **CHR** | **TEST** | **OR** | **STAT** | **P** |  | **TEST** | **OR** | **STAT** | **P** |
| RS1544325 | 1312 | COMT | 22 | ADD | 0.9899 | -0.1408 | 0.8880 |  | ADD | 1.0440 | 0.6272 | 0.5305 |
| RS165599 | 1312 | COMT | 22 | ADD | 1.0890 | 1.0520 | 0.2927 |  | ADD | 1.1360 | 1.6440 | 0.1003 |
| RS165656 | 1312 | COMT | 22 | ADD | 1.1130 | 1.4800 | 0.1389 |  | ADD | 0.9758 | -0.3572 | 0.7209 |
| RS165722 | 1312 | COMT | 22 | ADD | 1.1020 | 1.3470 | 0.1780 |  | ADD | 0.9773 | -0.3353 | 0.7374 |
| RS165728 | 1312 | COMT | 22 | ADD | 0.8962 | -0.6573 | 0.5110 |  | ADD | 0.8019 | -1.3890 | 0.1649 |
| RS165774 | 1312 | COMT | 22 | ADD | 0.9033 | -1.3130 | 0.1893 |  | ADD | 1.0080 | 0.1011 | 0.9195 |
| RS174675 | 1312 | COMT | 22 | ADD | 1.0110 | 0.1360 | 0.8918 |  | ADD | 0.8412 | -2.2150 | 0.0268 |
| RS174699 | 1312 | COMT | 22 | ADD | 0.8474 | -1.0040 | 0.3154 |  | ADD | 0.7985 | -1.4220 | 0.1550 |
| RS2239393 | 1312 | COMT | 22 | ADD | 1.1190 | 1.5050 | 0.1322 |  | ADD | 1.0120 | 0.1654 | 0.8686 |
| RS4646312 | 1312 | COMT | 22 | ADD | 1.1310 | 1.6500 | 0.0990 |  | ADD | 1.0140 | 0.1958 | 0.8448 |
| RS4646316 | 1312 | COMT | 22 | ADD | 1.0130 | 0.1532 | 0.8782 |  | ADD | 0.8682 | -1.8050 | 0.0711 |
| RS4680 | 1312 | COMT | 22 | ADD | 1.1010 | 1.3270 | 0.1845 |  | ADD | 0.9798 | -0.2970 | 0.7665 |
| RS5993883 | 1312 | COMT | 22 | ADD | 0.9854 | -0.2054 | 0.8373 |  | ADD | 1.0420 | 0.6059 | 0.5446 |
| RS737866 | 1312 | COMT | 22 | ADD | 0.9969 | -0.0374 | 0.9702 |  | ADD | 1.1150 | 1.3870 | 0.1655 |
| RS10993949 | 1621 | DBH | 9 | ADD | 0.8508 | -0.4343 | 0.6640 |  | ADD | 1.3400 | 0.7678 | 0.4426 |
| RS1108581 | 1621 | DBH | 9 | ADD | 1.0110 | 0.1207 | 0.9039 |  | ADD | 1.0730 | 0.7974 | 0.4252 |
| RS1541332 | 1621 | DBH | 9 | ADD | 0.9854 | -0.2016 | 0.8402 |  | ADD | 0.9765 | -0.3449 | 0.7302 |
| RS1611123 | 1621 | DBH | 9 | ADD | 1.0010 | 0.0142 | 0.9887 |  | ADD | 0.9706 | -0.4399 | 0.6600 |
| RS2007153 | 1621 | DBH | 9 | ADD | 1.0290 | 0.3768 | 0.7063 |  | ADD | 1.0100 | 0.1367 | 0.8913 |
| RS2097628 | 1621 | DBH | 9 | ADD | 1.1570 | 1.9230 | 0.0545 |  | ADD | 0.9274 | -1.0680 | 0.2855 |
| RS2283123 | 1621 | DBH | 9 | ADD | 1.3310 | 2.3680 | 0.0179 |  | ADD | 0.9957 | -0.0395 | 0.9685 |
| RS2283124 | 1621 | DBH | 9 | ADD | 1.3330 | 2.3830 | 0.0172 |  | ADD | 0.9974 | -0.0240 | 0.9809 |
| RS2519143 | 1621 | DBH | 9 | ADD | 0.9983 | -0.0180 | 0.9857 |  | ADD | 1.0270 | 0.3004 | 0.7639 |
| RS2519154 | 1621 | DBH | 9 | ADD | 0.9911 | -0.1225 | 0.9025 |  | ADD | 0.9796 | -0.3003 | 0.7639 |
| RS2519155 | 1621 | DBH | 9 | ADD | 0.9910 | -0.1191 | 0.9052 |  | ADD | 0.9715 | -0.4072 | 0.6838 |
| RS2797853 | 1621 | DBH | 9 | ADD | 1.0320 | 0.4070 | 0.6840 |  | ADD | 0.9868 | -0.1840 | 0.8540 |
| RS2873804 | 1621 | DBH | 9 | ADD | 0.9772 | -0.3208 | 0.7484 |  | ADD | 0.9853 | -0.2190 | 0.8267 |
| RS3025382 | 1621 | DBH | 9 | ADD | 1.0370 | 0.3106 | 0.7561 |  | ADD | 1.0460 | 0.4079 | 0.6834 |
| RS3025388 | 1621 | DBH | 9 | ADD | 1.0590 | 0.5679 | 0.5701 |  | ADD | 1.0220 | 0.2228 | 0.8237 |
| RS5320 | 1621 | DBH | 9 | ADD | 0.9929 | -0.0436 | 0.9653 |  | ADD | 0.8716 | -0.8751 | 0.3815 |
| RS77905 | 1621 | DBH | 9 | ADD | 0.9840 | -0.2256 | 0.8215 |  | ADD | 1.0560 | 0.8118 | 0.4169 |
| RS10268819 | 1644 | DDC | 7 | ADD | 0.9878 | -0.1078 | 0.9141 |  | ADD | 0.9448 | -0.5256 | 0.5991 |
| RS10499694 | 1644 | DDC | 7 | ADD | 0.9637 | -0.4972 | 0.6191 |  | ADD | 1.0730 | 0.9964 | 0.3190 |
| RS10499695 | 1644 | DDC | 7 | ADD | 0.9395 | -0.8432 | 0.3991 |  | ADD | 1.0600 | 0.8293 | 0.4069 |
| RS10499696 | 1644 | DDC | 7 | ADD | 1.0310 | 0.2715 | 0.7860 |  | ADD | 0.8888 | -1.1290 | 0.2587 |
| RS11238131 | 1644 | DDC | 7 | ADD | 0.9788 | -0.2687 | 0.7882 |  | ADD | 1.0080 | 0.1056 | 0.9159 |
| RS11238133 | 1644 | DDC | 7 | ADD | 0.9551 | -0.5920 | 0.5539 |  | ADD | 1.0340 | 0.4527 | 0.6507 |
| RS11575286 | 1644 | DDC | 7 | ADD | 0.9932 | -0.0592 | 0.9528 |  | ADD | 1.0640 | 0.5693 | 0.5691 |
| RS11575387 | 1644 | DDC | 7 | ADD | 1.0440 | 0.2985 | 0.7653 |  | ADD | 0.8951 | -0.8471 | 0.3969 |
| RS11575489 | 1644 | DDC | 7 | ADD | 0.8206 | -0.7154 | 0.4744 |  | ADD | 1.2220 | 0.7608 | 0.4468 |
| RS11575522 | 1644 | DDC | 7 | ADD | 1.1880 | 0.6210 | 0.5346 |  | ADD | 1.1570 | 0.5587 | 0.5764 |
| RS11575542 | 1644 | DDC | 7 | ADD | 1.1650 | 0.5074 | 0.6119 |  | ADD | 1.2330 | 0.7436 | 0.4571 |
| RS11768267 | 1644 | DDC | 7 | ADD | 0.9961 | -0.0531 | 0.9577 |  | ADD | 1.0280 | 0.4056 | 0.6850 |
| RS1349492 | 1644 | DDC | 7 | ADD | 0.9811 | -0.2591 | 0.7955 |  | ADD | 1.0030 | 0.0374 | 0.9702 |
| RS1376523 | 1644 | DDC | 7 | ADD | 0.9186 | -0.3871 | 0.6987 |  | ADD | 1.0670 | 0.3180 | 0.7505 |
| RS17133877 | 1644 | DDC | 7 | ADD | 0.9495 | -0.2705 | 0.7868 |  | ADD | 1.1730 | 0.8858 | 0.3757 |
| RS17634958 | 1644 | DDC | 7 | ADD | 1.0150 | 0.1377 | 0.8905 |  | ADD | 1.0380 | 0.3657 | 0.7146 |
| RS1966839 | 1644 | DDC | 7 | ADD | 0.9531 | -0.6195 | 0.5356 |  | ADD | 1.0970 | 1.2530 | 0.2103 |
| RS2329340 | 1644 | DDC | 7 | ADD | 0.9427 | -0.7628 | 0.4456 |  | ADD | 1.0990 | 1.2810 | 0.2002 |
| RS2329371 | 1644 | DDC | 7 | ADD | 0.9647 | -0.4038 | 0.6864 |  | ADD | 1.0760 | 0.8616 | 0.3889 |
| RS3735274 | 1644 | DDC | 7 | ADD | 0.9629 | -0.4527 | 0.6508 |  | ADD | 1.0130 | 0.1693 | 0.8656 |
| RS3779084 | 1644 | DDC | 7 | ADD | 1.0390 | 0.4387 | 0.6609 |  | ADD | 0.9844 | -0.1914 | 0.8482 |
| RS3807552 | 1644 | DDC | 7 | ADD | 0.9835 | -0.1924 | 0.8474 |  | ADD | 1.0840 | 0.9676 | 0.3333 |
| RS3807553 | 1644 | DDC | 7 | ADD | 0.9976 | -0.0211 | 0.9832 |  | ADD | 1.0580 | 0.5191 | 0.6037 |
| RS3807558 | 1644 | DDC | 7 | ADD | 0.9361 | -0.7444 | 0.4567 |  | ADD | 1.0310 | 0.3626 | 0.7169 |
| RS3829897 | 1644 | DDC | 7 | ADD | 0.9554 | -0.6054 | 0.5449 |  | ADD | 1.1330 | 1.7520 | 0.0798 |
| RS4947631 | 1644 | DDC | 7 | ADD | 0.7635 | -1.0330 | 0.3016 |  | ADD | 1.0920 | 0.3591 | 0.7195 |
| RS6592952 | 1644 | DDC | 7 | ADD | 0.9788 | -0.2910 | 0.7711 |  | ADD | 1.0060 | 0.0839 | 0.9331 |
| RS6592961 | 1644 | DDC | 7 | ADD | 1.0390 | 0.4407 | 0.6594 |  | ADD | 1.0060 | 0.0786 | 0.9373 |
| RS6593011 | 1644 | DDC | 7 | ADD | 0.9773 | -0.2279 | 0.8197 |  | ADD | 0.9518 | -0.5174 | 0.6048 |
| RS732215 | 1644 | DDC | 7 | ADD | 1.0190 | 0.2523 | 0.8008 |  | ADD | 1.0030 | 0.0375 | 0.9701 |
| RS7809758 | 1644 | DDC | 7 | ADD | 1.0180 | 0.2342 | 0.8149 |  | ADD | 0.9816 | -0.2648 | 0.7912 |
| RS880028 | 1644 | DDC | 7 | ADD | 1.0340 | 0.3806 | 0.7035 |  | ADD | 0.9836 | -0.2009 | 0.8408 |
| RS5326 | 1812 | DRD1 | 5 | ADD | 0.9072 | -0.9980 | 0.3183 |  | ADD | 1.1410 | 1.3930 | 0.1635 |
| RS686 | 1812 | DRD1 | 5 | ADD | 1.0230 | 0.3025 | 0.7623 |  | ADD | 0.9352 | -0.9626 | 0.3357 |
| RS1076563 | 1813 | DRD2 | 11 | ADD | 1.0260 | 0.3422 | 0.7322 |  | ADD | 1.0240 | 0.3338 | 0.7386 |
| RS1079727 | 1813 | DRD2 | 11 | ADD | 0.9511 | -0.4795 | 0.6315 |  | ADD | 0.9946 | -0.0540 | 0.9569 |
| RS11214606 | 1813 | DRD2 | 11 | ADD | 1.0590 | 0.3552 | 0.7224 |  | ADD | 0.9694 | -0.2036 | 0.8387 |
| RS17529477 | 1813 | DRD2 | 11 | ADD | 1.0090 | 0.1200 | 0.9045 |  | ADD | 0.9703 | -0.4147 | 0.6784 |
| RS2440390 | 1813 | DRD2 | 11 | ADD | 1.0440 | 0.4076 | 0.6835 |  | ADD | 1.0270 | 0.2742 | 0.7840 |
| RS2471857 | 1813 | DRD2 | 11 | ADD | 0.9556 | -0.4335 | 0.6647 |  | ADD | 1.0050 | 0.0523 | 0.9583 |
| RS2734838 | 1813 | DRD2 | 11 | ADD | 1.0300 | 0.3955 | 0.6925 |  | ADD | 1.0270 | 0.3771 | 0.7061 |
| RS4274224 | 1813 | DRD2 | 11 | ADD | 1.0220 | 0.3079 | 0.7582 |  | ADD | 0.9572 | -0.6446 | 0.5192 |
| RS4620755 | 1813 | DRD2 | 11 | ADD | 1.0800 | 0.6520 | 0.5144 |  | ADD | 1.0480 | 0.4163 | 0.6772 |
| RS4648317 | 1813 | DRD2 | 11 | ADD | 0.9484 | -0.5157 | 0.6061 |  | ADD | 1.0070 | 0.0691 | 0.9449 |
| RS4648318 | 1813 | DRD2 | 11 | ADD | 1.0040 | 0.0495 | 0.9606 |  | ADD | 1.0400 | 0.4956 | 0.6202 |
| RS4648319 | 1813 | DRD2 | 11 | ADD | 0.9392 | -0.6050 | 0.5452 |  | ADD | 1.0040 | 0.0413 | 0.9671 |
| RS4938019 | 1813 | DRD2 | 11 | ADD | 0.9755 | -0.2432 | 0.8078 |  | ADD | 1.0170 | 0.1772 | 0.8593 |
| RS7125415 | 1813 | DRD2 | 11 | ADD | 1.0620 | 0.4482 | 0.6540 |  | ADD | 1.0670 | 0.5095 | 0.6104 |
| RS7131056 | 1813 | DRD2 | 11 | ADD | 1.0240 | 0.3284 | 0.7426 |  | ADD | 1.0200 | 0.2789 | 0.7804 |
| RS10934256 | 1814 | DRD3 | 3 | ADD | 0.9556 | -0.4887 | 0.6251 |  | ADD | 0.9930 | -0.0790 | 0.9370 |
| RS11706283 | 1814 | DRD3 | 3 | ADD | 1.0020 | 0.0131 | 0.9895 |  | ADD | 0.8156 | -1.8180 | 0.0691 |
| RS1486009 | 1814 | DRD3 | 3 | ADD | 1.0160 | 0.1051 | 0.9163 |  | ADD | 0.9010 | -0.7255 | 0.4681 |
| RS167770 | 1814 | DRD3 | 3 | ADD | 0.9950 | -0.0617 | 0.9508 |  | ADD | 0.9739 | -0.3454 | 0.7298 |
| RS2134655 | 1814 | DRD3 | 3 | ADD | 0.9603 | -0.4815 | 0.6301 |  | ADD | 1.1170 | 1.3890 | 0.1649 |
| RS226082 | 1814 | DRD3 | 3 | ADD | 0.9904 | -0.1199 | 0.9045 |  | ADD | 0.9780 | -0.2904 | 0.7715 |
| RS2630349 | 1814 | DRD3 | 3 | ADD | 0.9808 | -0.1278 | 0.8983 |  | ADD | 0.9478 | -0.3692 | 0.7120 |
| RS2630351 | 1814 | DRD3 | 3 | ADD | 0.9769 | -0.1544 | 0.8773 |  | ADD | 0.9441 | -0.3957 | 0.6924 |
| RS324029 | 1814 | DRD3 | 3 | ADD | 0.9889 | -0.1382 | 0.8901 |  | ADD | 0.9783 | -0.2862 | 0.7747 |
| RS7633291 | 1814 | DRD3 | 3 | ADD | 0.9556 | -0.4887 | 0.6251 |  | ADD | 0.9930 | -0.0790 | 0.9370 |
| RS9288993 | 1814 | DRD3 | 3 | ADD | 1.3100 | 1.1390 | 0.2547 |  | ADD | 1.0600 | 0.2693 | 0.7877 |
| RS963468 | 1814 | DRD3 | 3 | ADD | 1.0660 | 0.8578 | 0.3910 |  | ADD | 0.9587 | -0.6038 | 0.5460 |
| DRD4R | 1815 | DRD4 | 11 | ADD | 0.8231 | -2.0090 | 0.0446 |  | ADD | 1.0510 | 0.5398 | 0.5894 |
| RS2617605 | 6531 | DAT | 5 | ADD | 1.1120 | 1.3820 | 0.1671 |  | ADD | 1.1230 | 1.6070 | 0.1081 |
| RS27048 | 6531 | DAT | 5 | ADD | 1.1000 | 1.3340 | 0.1822 |  | ADD | 1.0770 | 1.1050 | 0.2692 |
| RS27072 | 6531 | DAT | 5 | ADD | 1.1970 | 1.9200 | 0.0549 |  | ADD | 1.0500 | 0.5549 | 0.5789 |
| RS3776511 | 6531 | DAT | 5 | ADD | 0.8821 | -1.3980 | 0.1622 |  | ADD | 0.9815 | -0.2176 | 0.8277 |
| RS3776512 | 6531 | DAT | 5 | ADD | 0.8852 | -1.3580 | 0.1745 |  | ADD | 0.9846 | -0.1810 | 0.8563 |
| RS40184 | 6531 | DAT | 5 | ADD | 1.1350 | 1.7580 | 0.0787 |  | ADD | 1.0220 | 0.3270 | 0.7437 |
| RS403636 | 6531 | DAT | 5 | ADD | 0.9759 | -0.2421 | 0.8087 |  | ADD | 0.7873 | -2.5360 | 0.0112 |
| RS460000 | 6531 | DAT | 5 | ADD | 0.8766 | -1.4640 | 0.1432 |  | ADD | 0.9947 | -0.0621 | 0.9505 |
| RS460700 | 6531 | DAT | 5 | ADD | 0.8791 | -1.4310 | 0.1523 |  | ADD | 0.9974 | -0.0302 | 0.9759 |
| RS464049 | 6531 | DAT | 5 | ADD | 0.9567 | -0.6010 | 0.5478 |  | ADD | 1.0270 | 0.3899 | 0.6966 |
| RS6347 | 6531 | DAT | 5 | ADD | 0.8654 | -1.8080 | 0.0707 |  | ADD | 0.9398 | -0.8137 | 0.4158 |
| RS6350 | 6531 | DAT | 5 | ADD | 1.1800 | 0.9791 | 0.3276 |  | ADD | 1.1490 | 0.8824 | 0.3776 |
| RS6869645 | 6531 | DAT | 5 | ADD | 1.0650 | 0.4470 | 0.6549 |  | ADD | 0.9591 | -0.3129 | 0.7544 |
| RS12545707 | 6570 | VMAT1 | 8 | ADD | 0.9488 | -0.6257 | 0.5315 |  | ADD | 0.8893 | -1.4980 | 0.1341 |
| RS13258461 | 6570 | VMAT1 | 8 | ADD | 0.9739 | -0.3632 | 0.7165 |  | ADD | 1.0730 | 1.0140 | 0.3105 |
| RS1390938 | 6570 | VMAT1 | 8 | ADD | 1.0310 | 0.3658 | 0.7145 |  | ADD | 1.2180 | 2.4720 | 0.0134 |
| RS1390939 | 6570 | VMAT1 | 8 | ADD | 0.9987 | -0.0174 | 0.9861 |  | ADD | 1.0820 | 1.1310 | 0.2581 |
| RS1497020 | 6570 | VMAT1 | 8 | ADD | 1.0220 | 0.2896 | 0.7721 |  | ADD | 1.2220 | 2.7280 | 0.0064 |
| RS1497022 | 6570 | VMAT1 | 8 | ADD | 1.0330 | 0.4330 | 0.6650 |  | ADD | 0.9660 | -0.4810 | 0.6305 |
| RS1497023 | 6570 | VMAT1 | 8 | ADD | 1.0610 | 0.6142 | 0.5391 |  | ADD | 1.2650 | 2.5220 | 0.0117 |
| RS1497025 | 6570 | VMAT1 | 8 | ADD | 1.0840 | 0.8308 | 0.4061 |  | ADD | 1.2930 | 2.7370 | 0.0062 |
| RS2270637 | 6570 | VMAT1 | 8 | ADD | 0.9425 | -0.6324 | 0.5271 |  | ADD | 0.9245 | -0.8950 | 0.3708 |
| RS2270649 | 6570 | VMAT1 | 8 | ADD | 4.1060 | 1.3240 | 0.1855 |  | ADD | 1.1610 | 0.2083 | 0.8350 |
| RS2270650 | 6570 | VMAT1 | 8 | ADD | 1.0170 | 0.2224 | 0.8240 |  | ADD | 1.0130 | 0.1799 | 0.8572 |
| RS3779672 | 6570 | VMAT1 | 8 | ADD | 0.9384 | -0.6484 | 0.5167 |  | ADD | 0.8589 | -1.6380 | 0.1014 |
| RS4921692 | 6570 | VMAT1 | 8 | ADD | 0.8789 | -1.0200 | 0.3077 |  | ADD | 0.8331 | -1.5250 | 0.1273 |
| RS4922132 | 6570 | VMAT1 | 8 | ADD | 1.0730 | 0.7208 | 0.4710 |  | ADD | 1.3200 | 2.9220 | 0.0035 |
| RS6586896 | 6570 | VMAT1 | 8 | ADD | 1.0580 | 0.4565 | 0.6480 |  | ADD | 1.0930 | 0.7538 | 0.4510 |
| RS6586897 | 6570 | VMAT1 | 8 | ADD | 1.0080 | 0.1115 | 0.9112 |  | ADD | 1.0850 | 1.1560 | 0.2477 |
| RS6992927 | 6570 | VMAT1 | 8 | ADD | 1.0380 | 0.4587 | 0.6464 |  | ADD | 1.2170 | 2.4640 | 0.0137 |
| RS721950 | 6570 | VMAT1 | 8 | ADD | 1.0110 | 0.1403 | 0.8884 |  | ADD | 0.9594 | -0.5800 | 0.5619 |
| RS7841346 | 6570 | VMAT1 | 8 | ADD | 0.9263 | -0.8526 | 0.3939 |  | ADD | 0.8937 | -1.3310 | 0.1832 |
| RS952860 | 6570 | VMAT1 | 8 | ADD | 8.19e+08 | 0.0029 | 0.9977 |  | ADD | 3.0070 | 1.4080 | 0.1591 |
| RS10082463 | 6571 | VMAT2 | 10 | ADD | 0.8762 | -1.1000 | 0.2712 |  | ADD | 1.3460 | 2.4510 | 0.0142 |
| RS11197936 | 6571 | VMAT2 | 10 | ADD | 0.9713 | -0.3959 | 0.6922 |  | ADD | 1.0410 | 0.5681 | 0.5699 |
| RS1860404 | 6571 | VMAT2 | 10 | ADD | 1.1640 | 1.5000 | 0.1336 |  | ADD | 1.2360 | 2.1870 | 0.0288 |
| RS2015586 | 6571 | VMAT2 | 10 | ADD | 0.9051 | -1.3460 | 0.1784 |  | ADD | 0.9932 | -0.0985 | 0.9215 |
| RS2283138 | 6571 | VMAT2 | 10 | ADD | 0.9625 | -0.3300 | 0.7414 |  | ADD | 1.3210 | 2.4380 | 0.0148 |
| RS3523 | 6571 | VMAT2 | 10 | ADD | 1.0170 | 0.1610 | 0.8721 |  | ADD | 1.0790 | 0.7763 | 0.4376 |
| RS363221 | 6571 | VMAT2 | 10 | ADD | 1.1130 | 0.6902 | 0.4901 |  | ADD | 0.9716 | -0.1981 | 0.8430 |
| RS363224 | 6571 | VMAT2 | 10 | ADD | 0.9070 | -1.3280 | 0.1842 |  | ADD | 0.9859 | -0.2052 | 0.8374 |
| RS363225 | 6571 | VMAT2 | 10 | ADD | 0.9097 | -1.2860 | 0.1984 |  | ADD | 0.9682 | -0.4666 | 0.6408 |
| RS363227 | 6571 | VMAT2 | 10 | ADD | 0.9245 | -0.6922 | 0.4888 |  | ADD | 1.2130 | 1.7350 | 0.0828 |
| RS363230 | 6571 | VMAT2 | 10 | ADD | 1.0510 | 0.6820 | 0.4952 |  | ADD | 1.0730 | 1.0170 | 0.3092 |
| RS363251 | 6571 | VMAT2 | 10 | ADD | 1.0890 | 1.0980 | 0.2723 |  | ADD | 1.0970 | 1.2630 | 0.2067 |
| RS363275 | 6571 | VMAT2 | 10 | ADD | 1.0080 | 0.0739 | 0.9411 |  | ADD | 1.0810 | 0.7971 | 0.4254 |
| RS363276 | 6571 | VMAT2 | 10 | ADD | 0.9983 | -0.0165 | 0.9869 |  | ADD | 1.0720 | 0.7106 | 0.4773 |
| RS363332 | 6571 | VMAT2 | 10 | ADD | 1.0510 | 0.6245 | 0.5323 |  | ADD | 1.1170 | 1.4540 | 0.1460 |
| RS363341 | 6571 | VMAT2 | 10 | ADD | 1.0570 | 0.7036 | 0.4817 |  | ADD | 1.1090 | 1.3770 | 0.1685 |
| RS363387 | 6571 | VMAT2 | 10 | ADD | 1.0720 | 0.3438 | 0.7310 |  | ADD | 1.2260 | 1.0560 | 0.2908 |
| RS363397 | 6571 | VMAT2 | 10 | ADD | 1.0510 | 0.2473 | 0.8047 |  | ADD | 1.1960 | 0.9415 | 0.3464 |
| RS2070762 | 7054 | TH | 11 | ADD | 0.9566 | -0.6304 | 0.5284 |  | ADD | 0.9895 | -0.1592 | 0.8735 |
| RS6356 | 7054 | TH | 11 | ADD | 1.0110 | 0.1492 | 0.8814 |  | ADD | 1.0330 | 0.4599 | 0.6456 |
| RS1800497 | 255239 | ANKK1 | 11 | ADD | 1.0120 | 0.1307 | 0.8960 |  | ADD | 1.0070 | 0.0780 | 0.9378 |
| RS2734848 | 255239 | ANKK1 | 11 | ADD | 1.0360 | 0.3895 | 0.6969 |  | ADD | 1.1510 | 1.6280 | 0.1036 |
| RS2734849 | 255239 | ANKK1 | 11 | ADD | 1.0690 | 0.9283 | 0.3532 |  | ADD | 1.1040 | 1.4530 | 0.1462 |
| RS4590907 | 255239 | ANKK1 | 11 | ADD | 1.0080 | 0.0753 | 0.9399 |  | ADD | 0.9893 | -0.1043 | 0.9169 |
| RS7118900 | 255239 | ANKK1 | 11 | ADD | 1.0520 | 0.5243 | 0.6001 |  | ADD | 1.0230 | 0.2440 | 0.8072 |

***** TEST = Code for the test (ADD = additive effects of allele dosage)**

**OR = odds-ratio**

**STAT = Coefficient t-statistic**

**P = Asymptotic p-value for t-statistic**

***
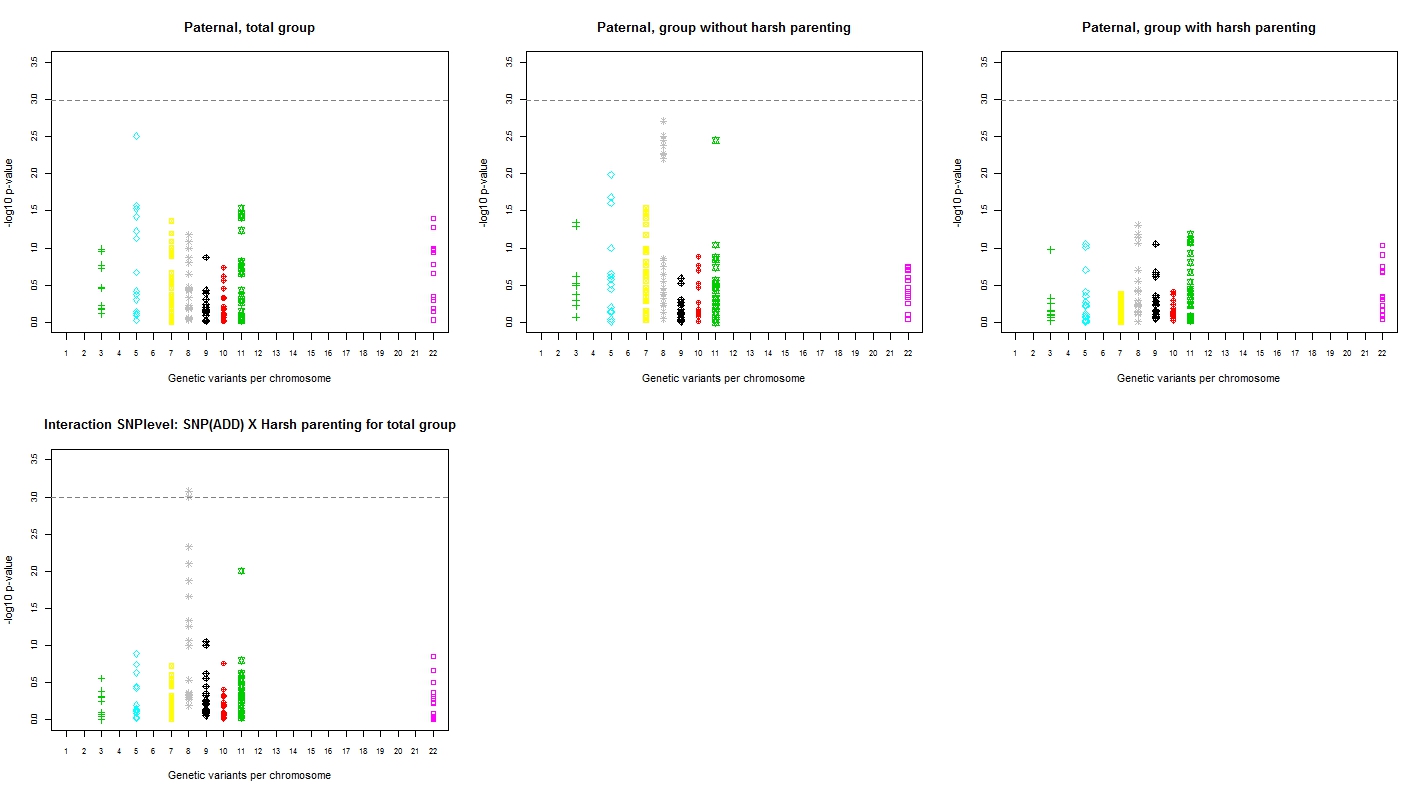
S5. Manhattan plots***

**A B C**

**D**

**A, B, C:** p-values for associations between single genetic variants and externalizing behavior for the total group and the groups without and with harsh parenting respectively. These p-values are used in JAG to compute the test-statistic for the gene-set analyses. Corresponding tables are presented in S6.

**D:** p-values for the interaction effect of SNP(ADD) and harsh parenting for the total group. Corresponding tables are presented in S8.

Dotted line represents significance threshold used at SNP-level, α = .0010.

***
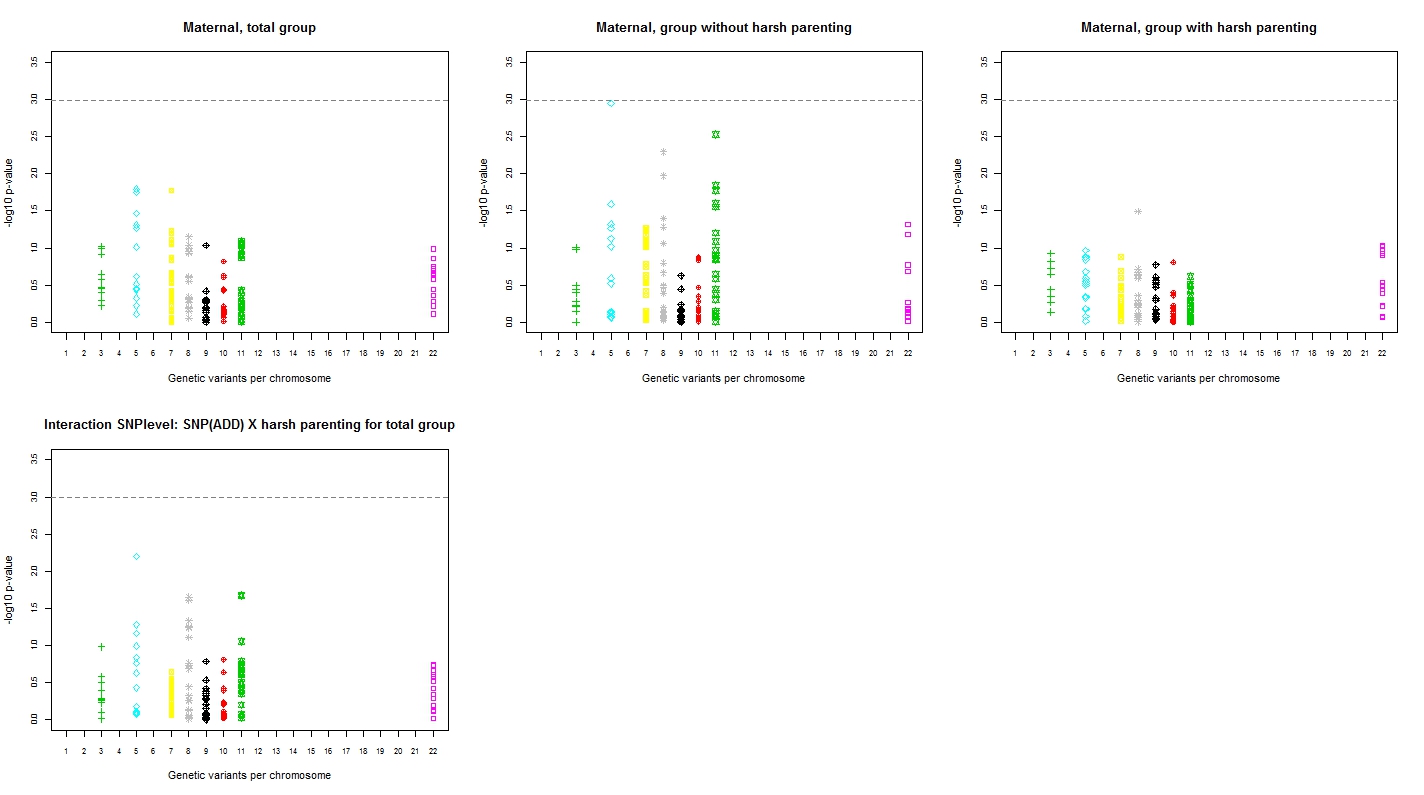
***

**A B C**

**D**

**A, B, C:** p-values for associations between single genetic variants and externalizing behavior for the total group and the groups without and with harsh parenting respectively. These p-values are used in JAG to compute the test-statistic for the gene-set analyses. Corresponding tables are presented in S6.

**D:** p-values for the interaction effect of SNP(ADD) and harsh parenting for the total group. Corresponding tables are presented in S8.

Dotted line represents significance threshold used at SNP-level, α = .0010.

**A, B, C:** p-values for associations between single genetic variants and externalizing behavior for the total group and the groups without and with harsh parenting respectively. These p-values are used in JAG to compute the test-statistic for the gene-set analyses. Corresponding tables are presented in S6.

**D:** p-values for the interaction effect of SNP(ADD) and harsh parenting for the total group. Corresponding tables are presented in S8.

Dotted line represents significance threshold used at SNP-level, α = .0010.

***S6. Tables with p-values for single SNP associations with externalizing behavior (TEST = ADD), from gene-set analyses – corresponding to Manhattan plots ABC (For paternal/maternal).*** **included covariates: 4 principal components of GWAS data, child age and gender*

|  |  |  |  | **Paternal** | | |  | **Maternal** | | |
| --- | --- | --- | --- | --- | --- | --- | --- | --- | --- | --- |
| **SNP** | **gene** | **gene** | **CHR** | **Total** | **Without harsh parenting** | **With harsh parenting** |  | **Total** | **Without harsh parenting** | **With**  **harsh parenting** |
| RS1544325 | 1312 | COMT | 22 | 0.1009 | 0.4095 | 0.1239 |  | 0.1027 | 0.0660 | 0.8678 |
| RS165599 | 1312 | COMT | 22 | 0.2202 | 0.3810 | 0.5928 |  | 0.5988 | 0.6470 | 0.3272 |
| RS165656 | 1312 | COMT | 22 | 0.1052 | 0.1839 | 0.4915 |  | 0.2196 | 0.7653 | 0.1035 |
| RS165722 | 1312 | COMT | 22 | 0.1028 | 0.1788 | 0.4670 |  | 0.2275 | 0.8284 | 0.0917 |
| RS165728 | 1312 | COMT | 22 | 0.9281 | 0.4369 | 0.6825 |  | 0.7675 | 0.9571 | 0.3642 |
| RS165774 | 1312 | COMT | 22 | 0.7105 | 0.5608 | 0.7903 |  | 0.2178 | 0.2071 | 0.6128 |
| RS174675 | 1312 | COMT | 22 | 0.1679 | 0.7808 | 0.0931 |  | 0.3640 | 0.8304 | 0.6003 |
| RS174699 | 1312 | COMT | 22 | 0.6464 | 0.2728 | 0.9175 |  | 0.5180 | 0.7625 | 0.2900 |
| RS2239393 | 1312 | COMT | 22 | 0.0402 | 0.1999 | 0.1773 |  | 0.1769 | 0.6784 | 0.1241 |
| RS4646312 | 1312 | COMT | 22 | 0.0532 | 0.2473 | 0.2081 |  | 0.1873 | 0.7228 | 0.1185 |
| RS4646316 | 1312 | COMT | 22 | 0.4550 | 0.9111 | 0.2077 |  | 0.4285 | 0.5328 | 0.3145 |
| RS4680 | 1312 | COMT | 22 | 0.1078 | 0.1957 | 0.4498 |  | 0.2362 | 0.8500 | 0.0958 |
| RS5993883 | 1312 | COMT | 22 | 0.1132 | 0.3411 | 0.2127 |  | 0.1363 | 0.1680 | 0.4079 |
| RS737866 | 1312 | COMT | 22 | 0.4992 | 0.4623 | 0.8192 |  | 0.2601 | 0.0479 | 0.8381 |
| RS10993949 | 1621 | DBH | 9 | 0.1340 | 0.4931 | 0.0889 |  | 0.0920 | 0.2352 | 0.2741 |
| RS1108581 | 1621 | DBH | 9 | 0.9470 | 0.7694 | 0.8255 |  | 0.9303 | 0.5805 | 0.5021 |
| RS1541332 | 1621 | DBH | 9 | 0.5763 | 0.7532 | 0.5217 |  | 0.8585 | 0.9034 | 0.8236 |
| RS1611123 | 1621 | DBH | 9 | 0.9619 | 0.4912 | 0.5873 |  | 0.9933 | 0.8620 | 0.7541 |
| RS2007153 | 1621 | DBH | 9 | 0.7953 | 0.9813 | 0.7213 |  | 0.8991 | 0.9942 | 0.8736 |
| RS2097628 | 1621 | DBH | 9 | 0.6764 | 0.7406 | 0.4396 |  | 0.4998 | 0.7121 | 0.6641 |
| RS2283123 | 1621 | DBH | 9 | 0.7442 | 0.2537 | 0.2291 |  | 0.5243 | 0.9642 | 0.3354 |
| RS2283124 | 1621 | DBH | 9 | 0.7108 | 0.2537 | 0.2115 |  | 0.4968 | 0.9642 | 0.3071 |
| RS2519143 | 1621 | DBH | 9 | 0.3664 | 0.9852 | 0.2420 |  | 0.5168 | 0.8093 | 0.1667 |
| RS2519154 | 1621 | DBH | 9 | 0.9561 | 0.9237 | 0.7910 |  | 0.6891 | 0.6959 | 0.9029 |
| RS2519155 | 1621 | DBH | 9 | 0.9550 | 0.6645 | 0.6877 |  | 0.9056 | 0.6886 | 0.7872 |
| RS2797853 | 1621 | DBH | 9 | 0.6232 | 0.8642 | 0.4667 |  | 0.6446 | 0.9741 | 0.4717 |
| RS2873804 | 1621 | DBH | 9 | 0.9290 | 0.4977 | 0.7111 |  | 0.8469 | 0.9143 | 0.9243 |
| RS3025382 | 1621 | DBH | 9 | 0.7963 | 0.5921 | 0.8700 |  | 0.6303 | 0.3576 | 0.7601 |
| RS3025388 | 1621 | DBH | 9 | 0.6825 | 0.9291 | 0.7001 |  | 0.5435 | 0.8355 | 0.2457 |
| RS5320 | 1621 | DBH | 9 | 0.4116 | 0.5310 | 0.5778 |  | 0.3811 | 0.6654 | 0.2884 |
| RS77905 | 1621 | DBH | 9 | 0.4854 | 0.2997 | 0.8924 |  | 0.7289 | 0.8005 | 0.9012 |
| RS10268819 | 1644 | DDC | 7 | 0.4528 | 0.3709 | 0.9333 |  | 0.2865 | 0.8083 | 0.2519 |
| RS10499694 | 1644 | DDC | 7 | 0.2631 | 0.1010 | 0.8626 |  | 0.2458 | 0.1641 | 0.7094 |
| RS10499695 | 1644 | DDC | 7 | 0.3427 | 0.1058 | 0.6589 |  | 0.3736 | 0.2872 | 0.7925 |
| RS10499696 | 1644 | DDC | 7 | 0.5635 | 0.5222 | 0.8431 |  | 0.4000 | 0.7333 | 0.6200 |
| RS11238131 | 1644 | DDC | 7 | 0.9225 | 0.9207 | 0.7581 |  | 0.8932 | 0.4322 | 0.4600 |
| RS11238133 | 1644 | DDC | 7 | 0.3057 | 0.1123 | 0.8235 |  | 0.2918 | 0.0801 | 0.7951 |
| RS11575286 | 1644 | DDC | 7 | 0.1087 | 0.0399 | 0.7536 |  | 0.1448 | 0.0593 | 0.8089 |
| RS11575387 | 1644 | DDC | 7 | 0.7225 | 0.4004 | 0.6857 |  | 0.9622 | 0.9032 | 0.8704 |
| RS11575489 | 1644 | DDC | 7 | 0.9447 | 0.5081 | 0.5271 |  | 0.9677 | 0.9093 | 0.7516 |
| RS11575522 | 1644 | DDC | 7 | 0.7536 | 0.7572 | 0.9942 |  | 0.6749 | 0.8967 | 0.7299 |
| RS11575542 | 1644 | DDC | 7 | 0.5843 | 0.8707 | 0.5661 |  | 0.6822 | 0.9389 | 0.6035 |
| RS11768267 | 1644 | DDC | 7 | 0.2749 | 0.1525 | 0.8692 |  | 0.2149 | 0.0539 | 0.7609 |
| RS1349492 | 1644 | DDC | 7 | 0.3090 | 0.1707 | 0.9454 |  | 0.2326 | 0.0872 | 0.8227 |
| RS1376523 | 1644 | DDC | 7 | 0.7310 | 0.7528 | 0.7997 |  | 0.4296 | 0.2985 | 0.9477 |
| RS17133877 | 1644 | DDC | 7 | 0.9986 | 0.7078 | 0.7711 |  | 0.9889 | 0.9435 | 0.7482 |
| RS17634958 | 1644 | DDC | 7 | 0.0634 | 0.0288 | 0.5518 |  | 0.0764 | 0.0669 | 0.4628 |
| RS1966839 | 1644 | DDC | 7 | 0.5068 | 0.2114 | 0.7076 |  | 0.6941 | 0.2490 | 0.6818 |
| RS2329340 | 1644 | DDC | 7 | 0.4853 | 0.2321 | 0.7408 |  | 0.5955 | 0.2312 | 0.8130 |
| RS2329371 | 1644 | DDC | 7 | 0.5248 | 0.7062 | 0.5606 |  | 0.4878 | 0.9439 | 0.3714 |
| RS3735274 | 1644 | DDC | 7 | 0.0813 | 0.0481 | 0.6774 |  | 0.0634 | 0.1781 | 0.2023 |
| RS3779084 | 1644 | DDC | 7 | 0.1190 | 0.1078 | 0.4196 |  | 0.0845 | 0.0927 | 0.5681 |
| RS3807552 | 1644 | DDC | 7 | 0.4219 | 0.6976 | 0.4346 |  | 0.5185 | 0.7813 | 0.3206 |
| RS3807553 | 1644 | DDC | 7 | 0.0998 | 0.0337 | 0.7536 |  | 0.1340 | 0.0593 | 0.7793 |
| RS3807558 | 1644 | DDC | 7 | 0.6563 | 0.9304 | 0.4651 |  | 0.6783 | 0.6979 | 0.3344 |
| RS3829897 | 1644 | DDC | 7 | 0.5482 | 0.2756 | 0.7239 |  | 0.6841 | 0.2936 | 0.8726 |
| RS4947631 | 1644 | DDC | 7 | 0.8876 | 0.4588 | 0.7921 |  | 0.8776 | 0.3798 | 0.4586 |
| RS6592952 | 1644 | DDC | 7 | 0.2944 | 0.1708 | 0.9185 |  | 0.2215 | 0.0785 | 0.8227 |
| RS6592961 | 1644 | DDC | 7 | 0.2138 | 0.2169 | 0.4918 |  | 0.1468 | 0.0895 | 0.8009 |
| RS6593011 | 1644 | DDC | 7 | 0.5922 | 0.3382 | 0.7621 |  | 0.4467 | 0.7471 | 0.6103 |
| RS732215 | 1644 | DDC | 7 | 0.1270 | 0.0666 | 0.7748 |  | 0.0578 | 0.0568 | 0.5204 |
| RS7809758 | 1644 | DDC | 7 | 0.0433 | 0.0329 | 0.4084 |  | 0.0168 | 0.0826 | 0.1316 |
| RS880028 | 1644 | DDC | 7 | 0.1227 | 0.1056 | 0.4502 |  | 0.0869 | 0.0977 | 0.5681 |
| RS5326 | 1812 | DRD1 | 5 | 0.2123 | 0.0207 | 0.8192 |  | 0.3487 | 0.0746 | 0.2526 |
| RS686 | 1812 | DRD1 | 5 | 0.9265 | 0.8988 | 0.9400 |  | 0.3048 | 0.8622 | 0.1075 |
| RS1076563 | 1813 | DRD2 | 11 | 0.0369 | 0.1820 | 0.0823 |  | 0.0890 | 0.0029 | 0.5483 |
| RS1079727 | 1813 | DRD2 | 11 | 0.0352 | 0.2982 | 0.0760 |  | 0.0811 | 0.0169 | 0.9851 |
| RS11214606 | 1813 | DRD2 | 11 | 0.9319 | 0.5328 | 0.5086 |  | 0.9875 | 0.8352 | 0.8575 |
| RS17529477 | 1813 | DRD2 | 11 | 0.1506 | 0.0905 | 0.8234 |  | 0.1252 | 0.0281 | 0.9537 |
| RS2440390 | 1813 | DRD2 | 11 | 0.4322 | 0.6100 | 0.3806 |  | 0.3922 | 0.1436 | 0.7411 |
| RS2471857 | 1813 | DRD2 | 11 | 0.0288 | 0.2717 | 0.0656 |  | 0.0857 | 0.0143 | 0.9572 |
| RS2734838 | 1813 | DRD2 | 11 | 0.0386 | 0.1820 | 0.0838 |  | 0.0922 | 0.0029 | 0.5387 |
| RS4274224 | 1813 | DRD2 | 11 | 0.8835 | 0.9216 | 0.8289 |  | 0.6013 | 0.1057 | 0.3742 |
| RS4620755 | 1813 | DRD2 | 11 | 0.8437 | 0.5973 | 0.3990 |  | 0.5612 | 0.7622 | 0.3672 |
| RS4648317 | 1813 | DRD2 | 11 | 0.2064 | 0.1335 | 0.9143 |  | 0.1062 | 0.1272 | 0.5073 |
| RS4648318 | 1813 | DRD2 | 11 | 0.3721 | 0.5323 | 0.4562 |  | 0.4728 | 0.0819 | 0.5166 |
| RS4648319 | 1813 | DRD2 | 11 | 0.0579 | 0.3689 | 0.1173 |  | 0.1327 | 0.0250 | 0.8899 |
| RS4938019 | 1813 | DRD2 | 11 | 0.2199 | 0.1431 | 0.8797 |  | 0.1051 | 0.1372 | 0.4466 |
| RS7125415 | 1813 | DRD2 | 11 | 0.4897 | 0.3356 | 0.9581 |  | 0.8009 | 0.3571 | 0.7231 |
| RS7131056 | 1813 | DRD2 | 11 | 0.9400 | 0.7792 | 0.5685 |  | 0.5712 | 0.7056 | 0.3050 |
| RS10934256 | 1814 | DRD3 | 3 | 0.1865 | 0.2378 | 0.4727 |  | 0.1221 | 0.5258 | 0.1181 |
| RS11706283 | 1814 | DRD3 | 3 | 0.3449 | 0.2996 | 0.8103 |  | 0.2680 | 0.3971 | 0.1859 |
| RS1486009 | 1814 | DRD3 | 3 | 0.1694 | 0.5052 | 0.1052 |  | 0.2252 | 0.3635 | 0.5309 |
| RS167770 | 1814 | DRD3 | 3 | 0.5945 | 0.4228 | 0.7777 |  | 0.3503 | 0.6160 | 0.3598 |
| RS2134655 | 1814 | DRD3 | 3 | 0.3381 | 0.3199 | 0.5556 |  | 0.3360 | 0.9883 | 0.2240 |
| RS226082 | 1814 | DRD3 | 3 | 0.6500 | 0.4228 | 0.7023 |  | 0.3943 | 0.7034 | 0.3598 |
| RS2630349 | 1814 | DRD3 | 3 | 0.1038 | 0.0454 | 0.8640 |  | 0.0961 | 0.0988 | 0.4488 |
| RS2630351 | 1814 | DRD3 | 3 | 0.1109 | 0.0505 | 0.8695 |  | 0.1003 | 0.1046 | 0.4478 |
| RS324029 | 1814 | DRD3 | 3 | 0.6623 | 0.4201 | 0.6839 |  | 0.3978 | 0.7110 | 0.3598 |
| RS7633291 | 1814 | DRD3 | 3 | 0.1865 | 0.2378 | 0.4727 |  | 0.1221 | 0.5258 | 0.1181 |
| RS9288993 | 1814 | DRD3 | 3 | 0.7608 | 0.8448 | 0.9539 |  | 0.5943 | 0.3208 | 0.7234 |
| RS963468 | 1814 | DRD3 | 3 | 0.6590 | 0.5965 | 0.7961 |  | 0.5065 | 0.5890 | 0.1520 |
| DRD4R | 1815 | DRD4 | 11 | 0.8267 | 0.9994 | 0.8317 |  | 0.5136 | 0.9837 | 0.2402 |
| RS2617605 | 6531 | DAT | 5 | 0.0744 | 0.0249 | 0.9712 |  | 0.0541 | 0.0011 | 0.2974 |
| RS27048 | 6531 | DAT | 5 | 0.4976 | 0.2401 | 0.5346 |  | 0.2411 | 0.0538 | 0.4663 |
| RS27072 | 6531 | DAT | 5 | 0.7950 | 0.9842 | 0.9860 |  | 0.4732 | 0.0961 | 0.2780 |
| RS3776511 | 6531 | DAT | 5 | 0.0269 | 0.2635 | 0.0888 |  | 0.0161 | 0.0478 | 0.1333 |
| RS3776512 | 6531 | DAT | 5 | 0.0296 | 0.2635 | 0.0972 |  | 0.0176 | 0.0478 | 0.1433 |
| RS40184 | 6531 | DAT | 5 | 0.3813 | 0.3596 | 0.3887 |  | 0.3588 | 0.8425 | 0.1311 |
| RS403636 | 6531 | DAT | 5 | 0.0031 | 0.0103 | 0.0971 |  | 0.0341 | 0.2541 | 0.2090 |
| RS460000 | 6531 | DAT | 5 | 0.7106 | 0.7030 | 0.6014 |  | 0.5979 | 0.7094 | 0.6641 |
| RS460700 | 6531 | DAT | 5 | 0.7127 | 0.7270 | 0.5849 |  | 0.5991 | 0.7316 | 0.6486 |
| RS464049 | 6531 | DAT | 5 | 0.4243 | 0.3115 | 0.7693 |  | 0.3639 | 0.3005 | 0.9588 |
| RS6347 | 6531 | DAT | 5 | 0.0379 | 0.2222 | 0.1985 |  | 0.0489 | 0.0753 | 0.3186 |
| RS6350 | 6531 | DAT | 5 | 0.0589 | 0.1002 | 0.4449 |  | 0.0968 | 0.0256 | 0.8312 |
| RS6869645 | 6531 | DAT | 5 | 0.7671 | 0.6219 | 0.8559 |  | 0.7701 | 0.7728 | 0.4471 |
| RS12545707 | 6570 | VMAT1 | 8 | 0.6371 | 0.2779 | 0.0493 |  | 0.6501 | 0.8034 | 0.2306 |
| RS13258461 | 6570 | VMAT1 | 8 | 0.6673 | 0.8899 | 0.5658 |  | 0.7072 | 0.7090 | 0.5140 |
| RS1390938 | 6570 | VMAT1 | 8 | 0.0824 | 0.0063 | 0.6104 |  | 0.0909 | 0.0522 | 0.8511 |
| RS1390939 | 6570 | VMAT1 | 8 | 0.6570 | 0.4200 | 0.7701 |  | 0.7013 | 0.1598 | 0.2309 |
| RS1497020 | 6570 | VMAT1 | 8 | 0.5929 | 0.1383 | 0.3878 |  | 0.4550 | 0.9437 | 0.6370 |
| RS1497022 | 6570 | VMAT1 | 8 | 0.9169 | 0.5893 | 0.6061 |  | 0.7029 | 0.8443 | 0.5633 |
| RS1497023 | 6570 | VMAT1 | 8 | 0.3358 | 0.0035 | 0.0711 |  | 0.2480 | 0.3347 | 0.9818 |
| RS1497025 | 6570 | VMAT1 | 8 | 0.1575 | 0.0031 | 0.1972 |  | 0.1126 | 0.3211 | 0.5814 |
| RS2270637 | 6570 | VMAT1 | 8 | 0.1347 | 0.0055 | 0.3659 |  | 0.1170 | 0.0106 | 0.5855 |
| RS2270649 | 6570 | VMAT1 | 8 | 0.2228 | 0.3457 | 0.0856 |  | 0.2419 | 0.7253 | 0.0321 |
| RS2270650 | 6570 | VMAT1 | 8 | 0.9137 | 0.5584 | 0.5852 |  | 0.7079 | 0.4065 | 0.8048 |
| RS3779672 | 6570 | VMAT1 | 8 | 0.3508 | 0.2259 | 0.9744 |  | 0.2491 | 0.8897 | 0.2118 |
| RS4921692 | 6570 | VMAT1 | 8 | 0.9233 | 0.7313 | 0.5771 |  | 0.8731 | 0.2149 | 0.2259 |
| RS4922132 | 6570 | VMAT1 | 8 | 0.3635 | 0.0053 | 0.0646 |  | 0.2808 | 0.4036 | 0.9039 |
| RS6586896 | 6570 | VMAT1 | 8 | 0.8978 | 0.4696 | 0.7044 |  | 0.4795 | 0.4049 | 0.7674 |
| RS6586897 | 6570 | VMAT1 | 8 | 0.3749 | 0.1483 | 0.7413 |  | 0.4950 | 0.0866 | 0.2558 |
| RS6992927 | 6570 | VMAT1 | 8 | 0.0664 | 0.0042 | 0.6011 |  | 0.0700 | 0.0399 | 0.9021 |
| RS721950 | 6570 | VMAT1 | 8 | 0.8625 | 0.3923 | 0.5111 |  | 0.6384 | 0.6362 | 0.6373 |
| RS7841346 | 6570 | VMAT1 | 8 | 0.1007 | 0.0020 | 0.2789 |  | 0.1003 | 0.0051 | 0.4330 |
| RS952860 | 6570 | VMAT1 | 8 | 0.4665 | 0.1779 | #NULL! |  | 0.5364 | 0.8316 | 0.1917 |
| RS10082463 | 6571 | VMAT2 | 10 | 0.9345 | 0.6595 | 0.8878 |  | 0.9711 | 0.9190 | 0.5917 |
| RS11197936 | 6571 | VMAT2 | 10 | 0.9267 | 0.3437 | 0.3826 |  | 0.7548 | 0.6239 | 0.9159 |
| RS1860404 | 6571 | VMAT2 | 10 | 0.9300 | 0.5470 | 0.7079 |  | 0.7372 | 0.4443 | 0.4379 |
| RS2015586 | 6571 | VMAT2 | 10 | 0.3536 | 0.3021 | 0.5051 |  | 0.2488 | 0.1464 | 0.7655 |
| RS2283138 | 6571 | VMAT2 | 10 | 0.7790 | 0.8322 | 0.7592 |  | 0.7313 | 0.7984 | 0.9817 |
| RS3523 | 6571 | VMAT2 | 10 | 0.6181 | 0.7574 | 0.7913 |  | 0.6783 | 0.7142 | 0.9274 |
| RS363221 | 6571 | VMAT2 | 10 | 0.2760 | 0.1304 | 0.8000 |  | 0.3770 | 0.5173 | 0.6466 |
| RS363224 | 6571 | VMAT2 | 10 | 0.4624 | 0.3402 | 0.6395 |  | 0.3554 | 0.1319 | 0.9366 |
| RS363225 | 6571 | VMAT2 | 10 | 0.4752 | 0.3030 | 0.7257 |  | 0.3669 | 0.1368 | 0.9792 |
| RS363227 | 6571 | VMAT2 | 10 | 0.7598 | 0.9718 | 0.7520 |  | 0.6015 | 0.8824 | 0.1543 |
| RS363230 | 6571 | VMAT2 | 10 | 0.1848 | 0.2014 | 0.4221 |  | 0.1508 | 0.1317 | 0.3942 |
| RS363251 | 6571 | VMAT2 | 10 | 0.2387 | 0.1732 | 0.9550 |  | 0.2348 | 0.3376 | 0.6364 |
| RS363275 | 6571 | VMAT2 | 10 | 0.6426 | 0.7794 | 0.7913 |  | 0.7017 | 0.6936 | 0.9997 |
| RS363276 | 6571 | VMAT2 | 10 | 0.6627 | 0.7892 | 0.7913 |  | 0.7232 | 0.7026 | 0.9997 |
| RS363332 | 6571 | VMAT2 | 10 | 0.8044 | 0.7390 | 0.8350 |  | 0.6717 | 0.9814 | 0.6365 |
| RS363341 | 6571 | VMAT2 | 10 | 0.9277 | 0.6757 | 0.5712 |  | 0.8518 | 0.8950 | 0.8364 |
| RS363387 | 6571 | VMAT2 | 10 | 0.9616 | 0.7602 | 0.7994 |  | 0.7700 | 0.8509 | 0.7045 |
| RS363397 | 6571 | VMAT2 | 10 | 0.8627 | 0.8430 | 0.7145 |  | 0.6842 | 0.9258 | 0.6003 |
| RS2070762 | 7054 | TH | 11 | 0.8594 | 0.4866 | 0.8482 |  | 0.9123 | 0.4985 | 0.6002 |
| RS6356 | 7054 | TH | 11 | 0.1733 | 0.0035 | 0.2855 |  | 0.1192 | 0.0627 | 0.8639 |
| RS1800497 | 255239 | ANKK1 | 11 | 0.1846 | 0.3453 | 0.3538 |  | 0.4698 | 0.2225 | 0.8534 |
| RS2734848 | 255239 | ANKK1 | 11 | 0.7138 | 0.7364 | 0.9277 |  | 0.7316 | 0.8164 | 0.9071 |
| RS2734849 | 255239 | ANKK1 | 11 | 0.5411 | 0.5455 | 0.5867 |  | 0.9800 | 0.4096 | 0.6693 |
| RS4590907 | 255239 | ANKK1 | 11 | 0.6994 | 0.6015 | 0.2111 |  | 0.6273 | 0.7033 | 0.3234 |
| RS7118900 | 255239 | ANKK1 | 11 | 0.1581 | 0.4673 | 0.1551 |  | 0.3701 | 0.2619 | 0.8423 |

***S7. Plots of test-statistic distributions of the original data and the permutations of the self-contained gene-set analyses in JAG.***

**Paternal harsh parenting, gene-set analyses**

Paternal, total group (n = 1710)

sum(-log10(p))= 72.9071, self-contained p: 0.2141

Paternal, group without harsh parenting (n = 1113)

sum(-log10(p)) = 88.1543, self-contained p: 0.025

Paternal, group with harsh parenting (n = 597)

sum(-log10(p)) = 47.2636, self-contained p: 0.9747

**Maternal harsh parenting, gene-set analyses**

Maternal, total group (n = 1881)

sum(-log10(p)) = 74.6457, self-contained p: 0.1909

Maternal, group without harsh parenting (n = 1208)

sum(-log10(p)) = 83.8715, self-contained p: 0.0533

Maternal, group with harsh parenting (n = 673)

sum(-log10(p)) = 50.1151, self-contained p: 0.9497

***S8. Tests of the interaction effect of SNP and harsh parenting (TEST = ADD X Harsh Parenting) for the total group (corresponding to Manhattan plots D)***

**total model consisted of: interaction (ADD X Harsh Parenting), ADD, Harsh Parenting, 4 principal components of GWAS data, child age and gender*

|  |  |  |  | **Paternal** | | |  | **Maternal** | | |
| --- | --- | --- | --- | --- | --- | --- | --- | --- | --- | --- |
| **SNP** | **gene** | **gene** | **CHR** | **BETA** | **STAT** | **P** |  | **BETA** | **STAT** | **P** |
| RS1544325 | 1312 | COMT | 22 | -0.2069 | -1.0040 | 0.3156 |  | 0.1772 | 0.8812 | 0.3783 |
| RS165599 | 1312 | COMT | 22 | -0.0323 | -0.1378 | 0.8904 |  | -0.2351 | -1.0400 | 0.2982 |
| RS165656 | 1312 | COMT | 22 | 0.0212 | 0.1022 | 0.9186 |  | -0.2473 | -1.2460 | 0.2128 |
| RS165722 | 1312 | COMT | 22 | 0.0160 | 0.0769 | 0.9387 |  | -0.2649 | -1.3320 | 0.1832 |
| RS165728 | 1312 | COMT | 22 | 0.3819 | 0.8005 | 0.4235 |  | -0.3475 | -0.7519 | 0.4522 |
| RS165774 | 1312 | COMT | 22 | 0.0097 | 0.0433 | 0.9655 |  | 0.0687 | 0.3226 | 0.7471 |
| RS174675 | 1312 | COMT | 22 | 0.3509 | 1.4760 | 0.1401 |  | 0.0646 | 0.2820 | 0.7780 |
| RS174699 | 1312 | COMT | 22 | 0.3317 | 0.7022 | 0.4827 |  | -0.3043 | -0.6619 | 0.5081 |
| RS2239393 | 1312 | COMT | 22 | -0.1184 | -0.5515 | 0.5814 |  | -0.2296 | -1.1190 | 0.2634 |
| RS4646312 | 1312 | COMT | 22 | -0.1143 | -0.5335 | 0.5937 |  | -0.2381 | -1.1660 | 0.2437 |
| RS4646316 | 1312 | COMT | 22 | -0.3005 | -1.2380 | 0.2161 |  | -0.1083 | -0.4761 | 0.6341 |
| RS4680 | 1312 | COMT | 22 | 0.0045 | 0.0216 | 0.9827 |  | -0.2659 | -1.3350 | 0.1821 |
| RS5993883 | 1312 | COMT | 22 | 0.1334 | 0.6480 | 0.5171 |  | -0.0084 | -0.0422 | 0.9663 |
| RS737866 | 1312 | COMT | 22 | -0.0513 | -0.2163 | 0.8287 |  | -0.2826 | -1.2330 | 0.2176 |
| RS10993949 | 1621 | DBH | 9 | -1.1680 | -1.0900 | 0.2757 |  | -0.5521 | -0.4988 | 0.6180 |
| RS1108581 | 1621 | DBH | 9 | -0.0811 | -0.3040 | 0.7611 |  | -0.1951 | -0.7705 | 0.4411 |
| RS1541332 | 1621 | DBH | 9 | -0.0704 | -0.3344 | 0.7382 |  | -0.0326 | -0.1621 | 0.8712 |
| RS1611123 | 1621 | DBH | 9 | -0.1950 | -0.9269 | 0.3541 |  | -0.0765 | -0.3868 | 0.6989 |
| RS2007153 | 1621 | DBH | 9 | 0.1093 | 0.4998 | 0.6173 |  | -0.0177 | -0.0850 | 0.9323 |
| RS2097628 | 1621 | DBH | 9 | 0.1254 | 0.5702 | 0.5686 |  | 0.0395 | 0.1909 | 0.8486 |
| RS2283123 | 1621 | DBH | 9 | -0.5642 | -1.6540 | 0.0983 |  | -0.2654 | -0.8273 | 0.4082 |
| RS2283124 | 1621 | DBH | 9 | -0.5836 | -1.7050 | 0.0884 |  | -0.2845 | -0.8843 | 0.3766 |
| RS2519143 | 1621 | DBH | 9 | 0.3210 | 1.1800 | 0.2383 |  | 0.3588 | 1.3940 | 0.1636 |
| RS2519154 | 1621 | DBH | 9 | -0.0550 | -0.2610 | 0.7941 |  | -0.0446 | -0.2237 | 0.8230 |
| RS2519155 | 1621 | DBH | 9 | -0.1599 | -0.7234 | 0.4695 |  | 0.0803 | 0.3890 | 0.6973 |
| RS2797853 | 1621 | DBH | 9 | 0.1189 | 0.5275 | 0.5979 |  | 0.1367 | 0.6494 | 0.5162 |
| RS2873804 | 1621 | DBH | 9 | 0.1592 | 0.7639 | 0.4450 |  | -0.0010 | -0.0053 | 0.9958 |
| RS3025382 | 1621 | DBH | 9 | -0.1203 | -0.3582 | 0.7203 |  | -0.2317 | -0.7180 | 0.4728 |
| RS3025388 | 1621 | DBH | 9 | -0.0663 | -0.2243 | 0.8226 |  | -0.2927 | -1.0530 | 0.2923 |
| RS5320 | 1621 | DBH | 9 | -0.0700 | -0.1497 | 0.8810 |  | -0.2866 | -0.6315 | 0.5278 |
| RS77905 | 1621 | DBH | 9 | 0.1271 | 0.6084 | 0.5430 |  | 0.0133 | 0.0677 | 0.9460 |
| RS10268819 | 1644 | DDC | 7 | -0.0791 | -0.2377 | 0.8122 |  | 0.2698 | 0.8661 | 0.3865 |
| RS10499694 | 1644 | DDC | 7 | -0.2330 | -1.0810 | 0.2800 |  | -0.0565 | -0.2739 | 0.7842 |
| RS10499695 | 1644 | DDC | 7 | -0.2823 | -1.3240 | 0.1857 |  | -0.0429 | -0.2107 | 0.8331 |
| RS10499696 | 1644 | DDC | 7 | 0.0089 | 0.0272 | 0.9783 |  | 0.0829 | 0.2736 | 0.7845 |
| RS11238131 | 1644 | DDC | 7 | -0.0799 | -0.3449 | 0.7302 |  | -0.2340 | -1.0750 | 0.2826 |
| RS11238133 | 1644 | DDC | 7 | -0.2603 | -1.1560 | 0.2480 |  | -0.2163 | -1.0050 | 0.3150 |
| RS11575286 | 1644 | DDC | 7 | -0.3024 | -0.9298 | 0.3526 |  | -0.2204 | -0.7005 | 0.4837 |
| RS11575387 | 1644 | DDC | 7 | 0.3881 | 0.9246 | 0.3553 |  | -0.0716 | -0.1889 | 0.8502 |
| RS11575489 | 1644 | DDC | 7 | -0.7541 | -0.9497 | 0.3424 |  | 0.2446 | 0.3198 | 0.7492 |
| RS11575522 | 1644 | DDC | 7 | 0.0980 | 0.1224 | 0.9026 |  | -0.1804 | -0.2385 | 0.8115 |
| RS11575542 | 1644 | DDC | 7 | -0.4259 | -0.4950 | 0.6207 |  | -0.4561 | -0.5584 | 0.5766 |
| RS11768267 | 1644 | DDC | 7 | -0.1419 | -0.6694 | 0.5033 |  | -0.2440 | -1.2040 | 0.2286 |
| RS1349492 | 1644 | DDC | 7 | -0.1514 | -0.7057 | 0.4805 |  | -0.2093 | -1.0290 | 0.3038 |
| RS1376523 | 1644 | DDC | 7 | -0.0893 | -0.1402 | 0.8885 |  | 0.3386 | 0.5745 | 0.5657 |
| RS17133877 | 1644 | DDC | 7 | 0.2468 | 0.4478 | 0.6543 |  | 0.1595 | 0.3062 | 0.7595 |
| RS17634958 | 1644 | DDC | 7 | -0.2192 | -0.7214 | 0.4707 |  | -0.0580 | -0.1961 | 0.8445 |
| RS1966839 | 1644 | DDC | 7 | -0.2574 | -1.1570 | 0.2473 |  | -0.1817 | -0.8487 | 0.3962 |
| RS2329340 | 1644 | DDC | 7 | -0.2396 | -1.0790 | 0.2807 |  | -0.1509 | -0.7050 | 0.4809 |
| RS2329371 | 1644 | DDC | 7 | -0.1145 | -0.4447 | 0.6566 |  | -0.1871 | -0.7580 | 0.4486 |
| RS3735274 | 1644 | DDC | 7 | -0.1523 | -0.6366 | 0.5245 |  | 0.0978 | 0.4298 | 0.6674 |
| RS3779084 | 1644 | DDC | 7 | -0.0272 | -0.1078 | 0.9142 |  | -0.0930 | -0.3871 | 0.6987 |
| RS3807552 | 1644 | DDC | 7 | -0.1494 | -0.5942 | 0.5525 |  | -0.2181 | -0.9077 | 0.3641 |
| RS3807553 | 1644 | DDC | 7 | -0.3146 | -0.9676 | 0.3334 |  | -0.2067 | -0.6579 | 0.5107 |
| RS3807558 | 1644 | DDC | 7 | -0.1678 | -0.6510 | 0.5151 |  | -0.2282 | -0.9224 | 0.3564 |
| RS3829897 | 1644 | DDC | 7 | -0.2307 | -1.0580 | 0.2902 |  | -0.1197 | -0.5747 | 0.5656 |
| RS4947631 | 1644 | DDC | 7 | 0.4959 | 0.6606 | 0.5089 |  | 0.7603 | 1.0840 | 0.2784 |
| RS6592952 | 1644 | DDC | 7 | -0.1438 | -0.6714 | 0.5021 |  | -0.2149 | -1.0560 | 0.2912 |
| RS6592961 | 1644 | DDC | 7 | -0.0109 | -0.0440 | 0.9649 |  | -0.1566 | -0.6540 | 0.5132 |
| RS6593011 | 1644 | DDC | 7 | -0.1887 | -0.6536 | 0.5135 |  | 0.0730 | 0.2648 | 0.7912 |
| RS732215 | 1644 | DDC | 7 | 0.1363 | 0.6353 | 0.5253 |  | 0.0791 | 0.3947 | 0.6931 |
| RS7809758 | 1644 | DDC | 7 | -0.0626 | -0.2935 | 0.7692 |  | 0.0726 | 0.3589 | 0.7197 |
| RS880028 | 1644 | DDC | 7 | -0.0410 | -0.1624 | 0.8710 |  | -0.0900 | -0.3748 | 0.7078 |
| RS5326 | 1812 | DRD1 | 5 | 0.4234 | 1.5200 | 0.1287 |  | 0.5333 | 1.9430 | 0.0522 |
| RS686 | 1812 | DRD1 | 5 | -0.0164 | -0.0778 | 0.9380 |  | -0.2738 | -1.3650 | 0.1724 |
| RS1076563 | 1813 | DRD2 | 11 | 0.1961 | 0.9198 | 0.3578 |  | -0.4677 | -2.3010 | 0.0215 |
| RS1079727 | 1813 | DRD2 | 11 | 0.3452 | 1.1510 | 0.2497 |  | -0.3727 | -1.2880 | 0.1979 |
| RS11214606 | 1813 | DRD2 | 11 | -0.4981 | -1.0760 | 0.2820 |  | -0.2139 | -0.4788 | 0.6321 |
| RS17529477 | 1813 | DRD2 | 11 | 0.1510 | 0.6815 | 0.4957 |  | 0.2743 | 1.2980 | 0.1943 |
| RS2440390 | 1813 | DRD2 | 11 | 0.1380 | 0.4587 | 0.6465 |  | -0.3345 | -1.1760 | 0.2397 |
| RS2471857 | 1813 | DRD2 | 11 | 0.3554 | 1.1830 | 0.2370 |  | -0.4041 | -1.3920 | 0.1640 |
| RS2734838 | 1813 | DRD2 | 11 | 0.1931 | 0.9066 | 0.3648 |  | -0.4698 | -2.3120 | 0.0209 |
| RS4274224 | 1813 | DRD2 | 11 | -0.0471 | -0.2286 | 0.8192 |  | 0.3356 | 1.7110 | 0.0872 |
| RS4620755 | 1813 | DRD2 | 11 | -0.3575 | -1.0370 | 0.3000 |  | -0.3758 | -1.1570 | 0.2476 |
| RS4648317 | 1813 | DRD2 | 11 | -0.2313 | -0.7910 | 0.4291 |  | -0.0599 | -0.2136 | 0.8309 |
| RS4648318 | 1813 | DRD2 | 11 | 0.0761 | 0.3124 | 0.7548 |  | -0.3915 | -1.7060 | 0.0882 |
| RS4648319 | 1813 | DRD2 | 11 | 0.3119 | 1.0460 | 0.2956 |  | -0.3867 | -1.3390 | 0.1808 |
| RS4938019 | 1813 | DRD2 | 11 | -0.2116 | -0.7273 | 0.4672 |  | -0.0278 | -0.0990 | 0.9212 |
| RS7125415 | 1813 | DRD2 | 11 | -0.1792 | -0.4589 | 0.6463 |  | -0.3682 | -1.0080 | 0.3138 |
| RS7131056 | 1813 | DRD2 | 11 | 0.1547 | 0.7353 | 0.4622 |  | -0.2634 | -1.3080 | 0.1911 |
| RS10934256 | 1814 | DRD3 | 3 | -0.0319 | -0.1195 | 0.9049 |  | -0.2575 | -1.0170 | 0.3093 |
| RS11706283 | 1814 | DRD3 | 3 | 0.0892 | 0.2591 | 0.7956 |  | -0.2133 | -0.6587 | 0.5102 |
| RS1486009 | 1814 | DRD3 | 3 | 0.4770 | 1.0860 | 0.2777 |  | 0.0188 | 0.0451 | 0.9641 |
| RS167770 | 1814 | DRD3 | 3 | 0.1347 | 0.5742 | 0.5659 |  | -0.1200 | -0.5404 | 0.5890 |
| RS2134655 | 1814 | DRD3 | 3 | 0.0018 | 0.0075 | 0.9940 |  | -0.2598 | -1.1250 | 0.2609 |
| RS226082 | 1814 | DRD3 | 3 | 0.1574 | 0.6706 | 0.5026 |  | -0.1356 | -0.6102 | 0.5418 |
| RS2630349 | 1814 | DRD3 | 3 | -0.3625 | -0.8282 | 0.4077 |  | -0.1161 | -0.2736 | 0.7844 |
| RS2630351 | 1814 | DRD3 | 3 | -0.3599 | -0.8222 | 0.4111 |  | -0.1136 | -0.2678 | 0.7889 |
| RS324029 | 1814 | DRD3 | 3 | 0.1636 | 0.6964 | 0.4863 |  | -0.1370 | -0.6162 | 0.5379 |
| RS7633291 | 1814 | DRD3 | 3 | -0.0319 | -0.1195 | 0.9049 |  | -0.2575 | -1.0170 | 0.3093 |
| RS9288993 | 1814 | DRD3 | 3 | 0.1431 | 0.2128 | 0.8315 |  | 0.5369 | 0.8437 | 0.3989 |
| RS963468 | 1814 | DRD3 | 3 | -0.0021 | -0.0097 | 0.9923 |  | 0.3311 | 1.6270 | 0.1038 |
| DRD4R | 1815 | DRD4 | 11 | -0.0210 | -0.0756 | 0.9397 |  | 0.3297 | 1.2160 | 0.2241 |
| RS2617605 | 6531 | DAT | 5 | 0.2946 | 1.3430 | 0.1795 |  | 0.5669 | 2.7350 | 0.0063 |
| RS27048 | 6531 | DAT | 5 | 0.2456 | 1.1950 | 0.2324 |  | 0.3193 | 1.6390 | 0.1013 |
| RS27072 | 6531 | DAT | 5 | 0.0276 | 0.1025 | 0.9184 |  | 0.4641 | 1.8210 | 0.0688 |
| RS3776511 | 6531 | DAT | 5 | 0.2383 | 0.9251 | 0.3550 |  | 0.0695 | 0.2794 | 0.7800 |
| RS3776512 | 6531 | DAT | 5 | 0.2287 | 0.8872 | 0.3751 |  | 0.0608 | 0.2444 | 0.8070 |
| RS40184 | 6531 | DAT | 5 | 0.0592 | 0.2873 | 0.7739 |  | 0.2332 | 1.1870 | 0.2353 |
| RS403636 | 6531 | DAT | 5 | -0.0206 | -0.0717 | 0.9429 |  | 0.1173 | 0.4290 | 0.6680 |
| RS460000 | 6531 | DAT | 5 | -0.0856 | -0.3270 | 0.7437 |  | -0.0537 | -0.2129 | 0.8315 |
| RS460700 | 6531 | DAT | 5 | -0.0949 | -0.3620 | 0.7174 |  | -0.0626 | -0.2479 | 0.8042 |
| RS464049 | 6531 | DAT | 5 | 0.0749 | 0.3541 | 0.7233 |  | 0.0895 | 0.4441 | 0.6570 |
| RS6347 | 6531 | DAT | 5 | 0.1092 | 0.4788 | 0.6321 |  | -0.0439 | -0.1979 | 0.8432 |
| RS6350 | 6531 | DAT | 5 | 0.0305 | 0.0620 | 0.9505 |  | 0.6692 | 1.4520 | 0.1466 |
| RS6869645 | 6531 | DAT | 5 | -0.0969 | -0.2404 | 0.8101 |  | 0.3451 | 0.8954 | 0.3707 |
| RS12545707 | 6570 | VMAT1 | 8 | -0.6011 | -2.4740 | 0.0134 |  | -0.2892 | -1.2660 | 0.2056 |
| RS13258461 | 6570 | VMAT1 | 8 | -0.0976 | -0.4579 | 0.6471 |  | -0.1424 | -0.7166 | 0.4737 |
| RS1390938 | 6570 | VMAT1 | 8 | 0.4565 | 1.9190 | 0.0552 |  | 0.3040 | 1.3210 | 0.1866 |
| RS1390939 | 6570 | VMAT1 | 8 | 0.1425 | 0.6728 | 0.5011 |  | 0.3804 | 1.9020 | 0.0574 |
| RS1497020 | 6570 | VMAT1 | 8 | 0.3601 | 1.6440 | 0.1004 |  | -0.0734 | -0.3410 | 0.7331 |
| RS1497022 | 6570 | VMAT1 | 8 | 0.1770 | 0.7949 | 0.4268 |  | -0.0696 | -0.3367 | 0.7364 |
| RS1497023 | 6570 | VMAT1 | 8 | 0.9155 | 3.3470 | 0.0008 |  | 0.1631 | 0.6049 | 0.5453 |
| RS1497025 | 6570 | VMAT1 | 8 | 0.7809 | 2.8330 | 0.0047 |  | 0.0113 | 0.0416 | 0.9668 |
| RS2270637 | 6570 | VMAT1 | 8 | -0.6224 | -2.2950 | 0.0219 |  | -0.4871 | -1.9160 | 0.0555 |
| RS2270649 | 6570 | VMAT1 | 8 | 5.2930 | 1.7170 | 0.0862 |  | 4.6570 | 2.2500 | 0.0245 |
| RS2270650 | 6570 | VMAT1 | 8 | 0.1599 | 0.7519 | 0.4522 |  | 0.1205 | 0.5960 | 0.5513 |
| RS3779672 | 6570 | VMAT1 | 8 | -0.2060 | -0.7250 | 0.4686 |  | 0.2454 | 0.9177 | 0.3589 |
| RS4921692 | 6570 | VMAT1 | 8 | -0.2283 | -0.6244 | 0.5325 |  | 0.6160 | 1.7650 | 0.0778 |
| RS4922132 | 6570 | VMAT1 | 8 | 0.9229 | 3.2990 | 0.0010 |  | 0.1672 | 0.6066 | 0.5442 |
| RS6586896 | 6570 | VMAT1 | 8 | -0.2587 | -0.7214 | 0.4707 |  | -0.0358 | -0.1029 | 0.9180 |
| RS6586897 | 6570 | VMAT1 | 8 | 0.2269 | 1.0540 | 0.2919 |  | 0.4053 | 1.9940 | 0.0463 |
| RS6992927 | 6570 | VMAT1 | 8 | 0.4749 | 1.9980 | 0.0459 |  | 0.3045 | 1.3280 | 0.1843 |
| RS721950 | 6570 | VMAT1 | 8 | 0.2327 | 1.0540 | 0.2918 |  | -0.0327 | -0.1596 | 0.8732 |
| RS7841346 | 6570 | VMAT1 | 8 | -0.6884 | -2.6580 | 0.0079 |  | -0.5607 | -2.2900 | 0.0222 |
| RS952860 | 6570 | VMAT1 | 8 | NA | NA | NA |  | 3.1020 | 1.3690 | 0.1713 |
| RS10082463 | 6571 | VMAT2 | 10 | 0.0943 | 0.2738 | 0.7843 |  | 0.1889 | 0.5362 | 0.5919 |
| RS11197936 | 6571 | VMAT2 | 10 | -0.2842 | -1.3630 | 0.1732 |  | -0.0284 | -0.1400 | 0.8887 |
| RS1860404 | 6571 | VMAT2 | 10 | -0.2030 | -0.6937 | 0.4880 |  | -0.3368 | -1.2040 | 0.2287 |
| RS2015586 | 6571 | VMAT2 | 10 | -0.0395 | -0.1874 | 0.8514 |  | 0.1109 | 0.5550 | 0.5790 |
| RS2283138 | 6571 | VMAT2 | 10 | -0.0749 | -0.2234 | 0.8232 |  | -0.0607 | -0.1821 | 0.8555 |
| RS3523 | 6571 | VMAT2 | 10 | -0.0505 | -0.1715 | 0.8639 |  | 0.0374 | 0.1314 | 0.8955 |
| RS363221 | 6571 | VMAT2 | 10 | -0.2463 | -0.5546 | 0.5792 |  | 0.0367 | 0.0879 | 0.9299 |
| RS363224 | 6571 | VMAT2 | 10 | -0.0089 | -0.0425 | 0.9661 |  | 0.1761 | 0.8880 | 0.3747 |
| RS363225 | 6571 | VMAT2 | 10 | 0.0211 | 0.1007 | 0.9198 |  | 0.1654 | 0.8325 | 0.4052 |
| RS363227 | 6571 | VMAT2 | 10 | 0.0566 | 0.1740 | 0.8619 |  | 0.4635 | 1.4200 | 0.1558 |
| RS363230 | 6571 | VMAT2 | 10 | 0.0376 | 0.1802 | 0.8570 |  | -0.0127 | -0.0644 | 0.9486 |
| RS363251 | 6571 | VMAT2 | 10 | 0.1589 | 0.7193 | 0.4720 |  | 0.0331 | 0.1575 | 0.8749 |
| RS363275 | 6571 | VMAT2 | 10 | -0.0543 | -0.1843 | 0.8538 |  | 0.0630 | 0.2211 | 0.8251 |
| RS363276 | 6571 | VMAT2 | 10 | -0.0563 | -0.1906 | 0.8489 |  | 0.0611 | 0.2139 | 0.8306 |
| RS363332 | 6571 | VMAT2 | 10 | 0.1029 | 0.4433 | 0.6576 |  | -0.0646 | -0.2948 | 0.7682 |
| RS363341 | 6571 | VMAT2 | 10 | 0.1955 | 0.8552 | 0.3926 |  | -0.0203 | -0.0943 | 0.9249 |
| RS363387 | 6571 | VMAT2 | 10 | -0.2700 | -0.4597 | 0.6458 |  | -0.2751 | -0.4934 | 0.6218 |
| RS363397 | 6571 | VMAT2 | 10 | -0.2890 | -0.5014 | 0.6162 |  | -0.2913 | -0.5317 | 0.5950 |
| RS2070762 | 7054 | TH | 11 | 0.1271 | 0.6289 | 0.5295 |  | 0.1494 | 0.7690 | 0.4420 |
| RS6356 | 7054 | TH | 11 | 0.5564 | 2.5810 | 0.0099 |  | 0.1742 | 0.8385 | 0.4018 |
| RS1800497 | 255239 | ANKK1 | 11 | 0.1077 | 0.4014 | 0.6882 |  | -0.2209 | -0.8608 | 0.3895 |
| RS2734848 | 255239 | ANKK1 | 11 | 0.0431 | 0.1664 | 0.8678 |  | 0.0471 | 0.1897 | 0.8495 |
| RS2734849 | 255239 | ANKK1 | 11 | 0.0300 | 0.1468 | 0.8833 |  | -0.1917 | -0.9726 | 0.3309 |
| RS4590907 | 255239 | ANKK1 | 11 | -0.4472 | -1.4110 | 0.1583 |  | -0.3566 | -1.2000 | 0.2304 |
| RS7118900 | 255239 | ANKK1 | 11 | 0.2602 | 0.9465 | 0.3440 |  | -0.1260 | -0.4788 | 0.6322 |

*** TEST = Code for the test (interaction effect ADD (additive effects of allele dosage) X Harsh Parenting (dichotomous))**

**BETA = Regression coefficient**

**STAT = Coefficient t-statistic**

**P = Asymptotic p-value for t-statistic**

***S9. Additional analyses: gene-set analyses with educational level***

Since the ‘no harsh parenting’ and ‘harsh parenting’ groups differed on various sample characteristics, additional analyses were conducted to investigate whether the gene-set results were specific for harsh parenting. First, two groups were made based on a dichotomous measure of educational level of the parents (‘high education’ if one or both or both parents completed high education, ‘no high education’ if none of the parents completed high education’). Information on parental educational level as an index of socio-economic status was obtained at child-age 5, by questionnaires. Similarly as for the harsh parenting groups, gene-set analyses were conducted on the total sample (n = 2216) and conditional on educational level. The self-contained *p*-values were .43 for the total sample, .18 for the ‘high education’ group and .79 for the ‘not high education’ group. In a next step, we examined whether the original results would be unchanged when the effect of educational level was taken out by residualizing parental educational level out of the harsh parenting sum scores by (Poisson) regression. Dichotomization of the residuals resulted in exactly the same groups as dichotomization of the original harsh parenting sum scores. This indicates that the group division (with and without harsh parenting) was not determined by differences in SES.
